# *Schistosoma mansoni* x *S. haematobium* hybrids frequently infecting sub-Saharan migrants in southeastern Europe: Egg DNA genotyping assessed by RD-PCR, sequencing and cloning

**DOI:** 10.1371/journal.pntd.0012942

**Published:** 2025-03-31

**Authors:** Alejandra De Elías-Escribano, Patricio Artigas, Joaquín Salas-Coronas, María Pilar Luzon-Garcia, Marta Reguera-Gomez, María Isabel Cabeza-Barrera, José Vázquez-Villegas, Jerôme Boissier, Santiago Mas-Coma, María Dolores Bargues

**Affiliations:** 1 Departamento de Parasitología, Facultad de Farmacia, Universidad de Valencia, Burjassot, Valencia, Spain; 2 Centro de Investigación Biomédica en Red de Enfermedades Infecciosas (CIBERINFEC), Instituto de Salud Carlos III, Madrid, Spain; 3 Tropical Medicine Unit, Hospital Universitario Poniente, El Ejido, Almería, Spain; 4 International Health Research Group of Almería (GISIA), Faculty of Health Sciences, University of Almería, La Cañada de San Urbano, Almería, Spain; 5 Tropical Medicine Unit, Distrito Sanitario Poniente de Almería, El Ejido, Almería, Spain; 6 IHPE, Univ. Montpellier, CNRS, IFREMER Université de Perpignan Via Domitia, Perpignan, France; University of Oxford, UNITED KINGDOM OF GREAT BRITAIN AND NORTHERN IRELAND

## Abstract

**Background:**

Globalization and neglected tropical diseases (NTDs) are increasingly closely linked. In recent years, Spain and Southern Europe are experiencing a considerable increase in the influx of migrants infected by NTDs, mainly from West African countries. This study focuses on imported schistosomiasis and the entry into Europe of hetero-specific hybrids between two human species, *Schistosoma mansoni* and *S. haematobium,* causing intestinal and urogenital schistosomiasis respectively.

**Methodology/principal findings:**

Individualized genetic identification by molecular analysis using RD-PCR, sequencing and cloning of nuclear rDNA and mtDNA of 134 *Schistosoma* eggs was performed, including 41 lateral-spined and 84 terminal-spined eggs from urine, and nine lateral-spined eggs from stools. These eggs were recovered from six migrant males from Senegal, Guinea-Bissau, Côte d’Ivoire and Mali, who shared ectopic shedding of *S. mansoni*-like eggs in their urine. A high hybridization complexity was detected in the eggs of these patients, involving three *Schistosoma* species. The six patients were infected by *S. mansoni x S. haematobium* hybrids shedding *S. mansoni*-like eggs, and also *S. haematobium x S. curassoni* hybrids shedding *S. haematobium-*like eggs. *SmxSh* hybrids were mostly detected in *S. mansoni*-like eggs from urine (94.59%), whereas in feces the detection of those hybrids was less frequent (5.41%).

**Conclusions/significance:**

This study contributes to: (i) a better understanding of the heterospecific hybrids between *S. mansoni* and *S. haematobium* from the genetic point of view; (ii) it shows the frequency with which they are entering non-endemic countries, such as Spain and consequently in Europe; (iii) it determines the diversity of hybrid eggs and haplotypes that can occur within a single patient, e.g., up to two types of hybrids involving three *Schistosoma* species and up to six different haplotypes; (iv) it provides information to be considered in clinical presentations, diagnosis, responses to treatment and epidemiological impact in relation to possible transmission and establishment in non-endemic areas.

## Author summary

This study highlights unexpected genetic characteristics of imported schistosomiasis in non-endemic areas of Europe, mainly focusing on hybrids between the non-phylogenetically related human species *Schistosoma mansoni* and *S. haematobium*, which cause intestinal or urogenital schistosomiasis, respectively. The genetic identification, by molecular analysis of each individualized egg in urine, was performed on six migrant males from Senegal, Guinea-Bissau, Côte d’Ivoire, and Mali diagnosed with urogenital schistosomiasis in Spain. Genetic scrutiny was applied to both lateral-spined and terminal-spined schistosome eggs allowing to corroborate the frequent hybridization of *S. mansoni x S. haematobium*, but also the more common *S. haematobium x S. curassoni* hybrids, in addition to the simultaneous presence of hybrid eggs involving three *Schistosoma* species in the urine of the same patient, including *SmxSh* in *S. mansoni*-like eggs and *ShxSc* or *ShxSb/Sc* in *S. haematobium-*like eggs. This study genetically characterizes the hybrids between *S. mansoni* and *S. haematobium*, their presence in migrants living in non-endemic countries, and the diversity of hybrid types and haplotypes, including even up to six different haplotypes within a single patient. Results highlight the need to assess differences in the clinical picture, diagnosis, treatment responses, and the epidemiological impact regarding the potential transmission and establishment in non-endemic areas.

## Introduction

Schistosomiasis is caused by trematode worms of the genus *Schistosoma* and is included within neglected tropical diseases (NTDs). Approximately 779 million people reside in regions where this disease is endemic [1] and at least 251.4 million people required preventive chemotherapy in 2021 [2,3]. Local visitors and international tourists to these regions can also acquire schistosomiasis. These travel-related infections are an important but frequently overlooked global public health concern.

About 93% of all schistosomiasis cases are found in sub-Saharan Africa [4]. As a consequence of the migratory flow from African countries to Europe in recent years, there is a considerable increase of the number of patients diagnosed with schistosomiasis in non-endemic regions [5–12]. The prevalence of infection in sub-Saharan migrants living in such non-endemic countries has been estimated at 24% [13].

Of particular concern are the recent cases of autochthonous transmission reported in susceptible European Mediterranean regions were the combination of both the presence of a competent intermediate host and the chance of schistosomes being imported by migrants and travelers returning from sub-Saharan Africa coexist, as are the cases of Corsica Island in France [14–18] and the Poniente Area in Spain [19].

Hybridization phenomena between *Schistosoma* species are also particularly worrisome and an emerging public health concern. Although several *Schistosoma* species are host-specific and geographically separated, which maintains barriers between species and avoids their encounter. It should be considered that hetero-specific crosses between species may occur during the sexual stage within the mammalian host. Some of these natural hybridizations are prevalent across much of sub-Saharan Africa [20,21]. Genetic interactions occurring between the human urogenital schistosome species *S. haematobium* and closely-related intestinal schistosome species of livestock, such as *S. bovis, S. curassoni*, and *S. mattheei*, evidence the potential major public health impact due to their zoonotic features [22–24]. Data indicate that hybrids within the *haematobium* group of species with terminal-spined eggs are particularly common in West and Central Africa [25].

*Schistosoma haematobium* and *S. mansoni* are responsible for the majority of human schistosomiasis infections in Africa. These species share host specificity for humans, but their tropisms within their definitive hosts are different causing two pathological profiles, urogenital schistosomiasis and intestinal schistosomiasis, respectively. In the former, hematuria is the most common symptom and may develop severe bladder, kidney, ureteral and genital pathologies [26,27]. The latter is associated with mild intestinal symptoms and bloody diarrhea with the risk of causing severe morbidity with hepatosplenomegaly [28,29]. The morphology of the eggs also differs between these two species. The eggs of *S. haematobium* are expected to be detected in urine and are characterized by a terminal spine, whereas the eggs of *S. mansoni* are expected to be detected in the stool and have a lateral spine [30].

These two *Schistosoma* species are co-endemic in 35 African countries [31,32] and can potentially coinfect the same human host [33–36]. Thus, mixed infections have been frequently documented in different countries of Africa [33–39]. In regions where the disease is endemic, the coexistence of vector snails (*Biomphalaria* spp. for *S. mansoni* and *Bulinus* spp. for *S. haematobium*) together with the existence of both schistosome species in the same water collection underlies the risk of coinfection [31,40,41] and hybridization [42]. Furthermore, in some endemic countries such as Nigeria [39], Senegal [34], and Cameroon [43], the presence of lateral-spined eggs typical of *S. mansoni* in human urine and also terminal-spined eggs typical of *S. haematobium* in stool have been reported. The high frequency of ectopic *S. mansoni*-like eggs in urine and *S. haematobium-*like eggs in stool has been suggested to be due to either interspecific competition between the two schistosomes or a spillover from high infection loads [43].

The high phylogenetic distance between them evidences the belonging of these two species to two different evolutionary lineages, the *S. mansoni* and *S. haematobium* groups respectively. Physiological barriers in the adult’s location within the definitive host would prevent heterospecific crosses with a viable offspring [20,44]. Therefore, the recent genetically-documented reports of *S. haematobium* x *S. mansoni* hybrids in seven individual eggs/miracidia from school children in Senegal [42] and in two migrants diagnosed in France shedding ectopic *S. mansoni*-like eggs from Côte d’Ivoire [45,46], were pronouncedly unexpected.

Considering the movements of travelers and migrants within a globalized world, and the current global warming in which temperature may significantly affect the schistosome life-cycle and the survival/spread of the intermediate snail hosts [47], it is evident that the study of these hybrids may be crucial in the way to understand their epidemiology and prevent its potential widespread. Transmission of other species such as *S. mansoni* in Europe has not been documented so far. Nevertheless, *Biomphalaria tenagophila,* which is involved in the transmission of *S. mansoni* in Brazil, has been identified in Europe (Romania) [48] and given the numerous reports of *S. mansoni* infections in migrants and tourists visiting Romania and neighboring Hungary, there may be a potential risk of local transmission of intestinal schistosomiasis [49]. Moreover, other susceptible snail species, such as *B. glabrata, B. straminea, B. tenagophila* and *B. pfeifferi*, have proven their capacity to invade new latitudes and continents [50–52]. Similarly, as in lymnaeid vectors of fascioliasis [53], a *Biomphalaria* spp. snail self-fertilizes and may, therefore, act as a founder of an entire colony facilitating not only the potential transmission of *S. mansoni* but also its spread throughout non-endemic areas.

In the present study, we report the co-detection of lateral-spined schistosome eggs (typical of *S. mansoni*) and terminal-spined schistosome eggs (typical of *S. haematobium*) in urine samples from migrant males from Senegal, Guinea-Bissau, Côte d’Ivoire and Mali, previously diagnosed with urogenital schistosomiasis in Spain. The molecular characterization of both types of eggs, by nuclear rDNA/mtDNA genotyping using Rapid Diagnostics by PCR (RD-PCR), sequencing and cloning, allows us to corroborate not only the frequent hybridization of *S. mansoni* x *S. haematobium*, as well as the simultaneous presence of *S. haematobium* x *S. curassoni* hybrids in migrant patients living in Southern Europe (Spain).

The hybridization phenomenon between these genetically distant species provides new knowledge about the schistosome species and types of hybrids that enter with migrants. Such findings imply potential repercussions not only in the clinical picture, diagnosis and treatment response, but also in the epidemiological connotations that they could have in non-endemic areas where both infected humans and susceptible snails currently coexist [19,54–56].

## Materials and methods

### Ethics statement

This study has been approved by the Ethics Committee of the Hospital Poniente (protocol Schis-01-UMT-2018). Procedures were performed in accordance with the ethical standards laid down in the Declaration of Helsinki as revised in 2013. The informed consent form was signed by all participants enrolled in the study. All patients were invited to learn about their parasitological results, and participants found positive for schistosomiasis were offered free treatment (single 40 mg/kg dose of praziquantel). All patients included in this study responded to a single dose of praziquantel [57], except one who needed a second dose after six months.

### Samples

**Fig 1:**
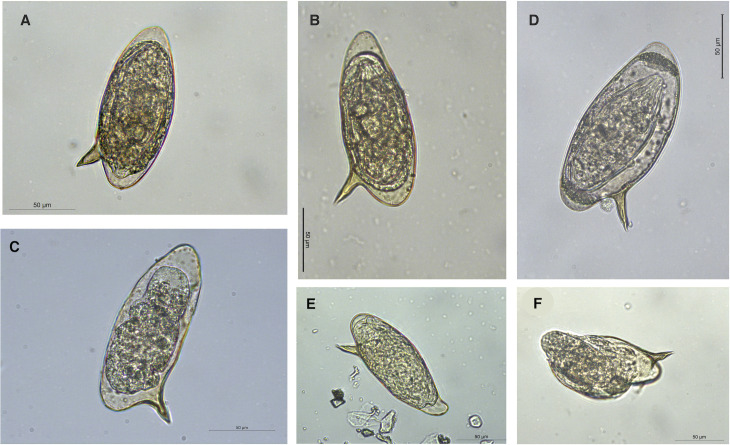
Photographs illustrating the morphology of *Schistosoma mansoni*-like eggs shed by the urine of migrant patients residing in Spain. A–C: from 3 different patients from Senegal; D: from a patient from Ivory Coast; E: from a patient from Guinea-Bissau; F: miracidium hatching under microscope light, in one egg from Senegal. (optical microscopy, original magnification, A–F = ×400; no stain used). Scale bars: A–F = 50 μm.

This study analyzes a total of 134 *Schistosoma* eggs. These include 41 lateral-spined *S. mansoni*-like eggs and 84 terminal-spined *S. haematobium*-like eggs recovered from urine samples, as well as nine lateral-spined *S. mansoni*-like eggs obtained from stool samples (Fig 1 and Table 1). The eggs were collected from six migrant males, who all exhibited shedding of *S. mansoni*-like eggs in their urine. These patients were young men, aged between 19 and 25 years, who had lived in Spain for between three and 48 months. None of them mentioned to have returned to an endemic area of schistosomiasis. Three migrant patients were from Senegal, one from Guinea-Bissau (this patient reported bathing activities in the Senegal River), one from Côte d’Ivoire, and one from Mali.

**Table 1:** *S. mansoni* (*Sm*) and *S. haematobium* (*Sh*) like-eggs processed, according to their shedding way (urine or stool) and patient country of origin.

Patient code	Years	Gender	Country	Egg morphology			
				*Sm*-like	*Sh*-like	*Sm*-like	Total
				Urine	Urine	Stool	
1Se	25	Male	Senegal	3	7	9	19
2Se	19	Male	Senegal	16	24	–	40
3Se	23	Male	Senegal	10	9	–	19
1Gb	21	Male	Guinea-Bissau	2	22	–	24
1 Ci	23	Male	Côte d’Ivoire	2	13	–	15
1Ma	21	Male	Mali	8	9	–	17
**Total**				41	84	9	**134**

The six patients included in the present study were previously diagnosed and confirmed with schistosomiasis at the Tropical Medicine Unit (TMU) of the Poniente University Hospital (El Ejido, Almería, Spain). After obtaining their consent, urine and stool samples were analyzed in the Parasitology Sanitary Unit of Valencia, Spain. These six patients represented the 12.24% (6/49) of migrant people diagnosed with schistosomiasis at the TMU in the period of April 2018-July 2024.

### Filtration of urine and stool samples

Only urine or fecal samples from patients with positive confirmation by egg findings were included in this study. Parasite eggs were recovered by individually filtering the total volume (30–210 mL, average 126.7 mL) of each of the six urine samples by using a new sterilized nylon cell strainer with a pore size of 40 µm (Falcon, Durham, NC, USA) for each sample. After filtration, the strainer was then rinsed with a 0.9% NaCl saline solution, to recover, in a Petri dish, any eggs that may have been trapped. The only positive stool sample, from one Senegalese patient, was appropriately filtered using the same saline solution through a column of metal sieves, stacked in decreasing order of pore size (0.5 mm, 0.25 mm, 0.125 mm, 0.1 mm and 0.04 mm) (FILTRA, Barcelona, Spain), to isolate parasite eggs from the debris. The filtrate from the last sieve (0.04 mm) was recovered on a Petri dish and used to examine the presence of *Schistosoma* eggs.

After filtration of urine and stool samples, all recovered eggs were collected one by one with a pipette of 10 μL from the Petri dishes under a stereomicroscope and each egg was placed in an Eppendorff vial containing 50 µL of 70% ethanol, for subsequent individual molecular characterization. All *S. mansoni* and *S. haematobium* like-eggs processed for molecular analyses are listed in Table 1.

### Molecular analyses

#### DNA extraction.

The 134 *Schistosoma* eggs (50 lateral-spined eggs and 84 terminal-spined eggs) recovered from the six urine and one stool samples were individually used for genetic characterization (Table 1). Genomic DNA was extracted individually from each egg - after allowing ethanol to evaporate naturally at room temperature until a residue of approximately 2 µL remained. The extraction was carried out using 115 µL of the InstaGene Matrix kit, made with a specially formulated 6% w/v Chelex resin (Bio-Rad Laboratories CA, USA), following the manufacturer’s instructions (https://www.bio-rad.com). DNA in the supernatant (90–95 µL) is ready for PCR amplification or can be stored at −20 °C until use.

#### RD-PCR of the cytochrome c oxidase subunit I (*cox*1).

For mitochondrial profiling, a rapid diagnostic multiplex one-step polymerase chain reaction (RD-PCR) from each of the 134 *Schistosoma* eggs was performed using species-specific primers [58–60] to amplify a specific length-differing region of the mtDNA *cox*1 gene for *S. bovis* (306 bp), *S. mansoni* (375 bp) and *S. haematobium* (543 bp) (S1 Appendix). The PCR reactions were performed in a final volume of 12.6 μL and comprising 8 μL of DNA template, as previously described [58,61]. The PCR conditions were 3 min at 95 °C, followed by 35 cycles of 30 secs at 94 °C, 1.30 min at 58 °C and 1.30 min at 72 °C followed by a final cycle at 72 °C for 7 min [60–62].

The *cox*1 RD-PCR products, along with positive controls (*S. haematobium, S. mansoni, S. bovis*), a negative control (water), and a 100 bp size standard ladder, were visualized by electrophoresis (30 min at 135V) on a 2.5% agarose gel stained with 5 μL of GelRed (Biotium, San Francisco, CA, USA). After electrophoresis, gels were photographed using the UVP gel documentation system (S2 Appendix).

#### Nuclear rDNA and mtDNA amplification and sequencing.

The information provided by the maternally inherited mtDNA profiling by RD-PCR was complemented with the partial sequence of the 5.8S gene (139 bp) and the complete internal transcribed spacer 2 (ITS-2) (314 bp) of the nuclear ribosomal DNA, to create a mito-nuclear signature (*cox*1/ITS-2). rDNA amplification by PCR was independently performed on each of the 134 eggs using primers 3S and A28S [60], which target the flanking regions of the 5.8S and 28S rRNA genes (S1 Appendix). Each PCR is made up of 11.2 μL of ultra-pure water, 5 μL of dNTPs mix, 10 mM each (Fisher Scientifc, Madrid, Spain), 0.5 μL of 10 μM of each primer, 2.5 μL of buffer (10× reaction buffer MgCl_2_ free), 2 μL of 2 mM MgCl_2_, 0.3 μL of 5 U/ μL of DNA Polymerase (Biotools, Madrid, Spain), and 8 μL of DNA extract, for a total volume of 30 μL for the PCR mix. PCR conditions were an activation step of 4 min at 94 °C, continued by 32 cycles of 55 secs at 94 °C, 1 min at 55–62 °C and 1.30 min at 72 °C each and a final extension of 5 min at 72 °C followed by a final cooling at 4 °C. Eight microliters of each amplicon were run out on agarose gels and photographed, as described above.

Additional amplification and sequencing of the 5’ end of the nuclear ribosomal 18S DNA gene (1369 bp) was performed in five eggs, in which double peaks were observed in the four polymorphic positions of the ITS-2 sequence that do not distinguish between *S. bovis* (*Sb*) and *S. curassoni* (*Sc*). These procedures were performed as previously described [63].

Of the 134 eggs from which both *cox*1 (short fragment, RD-PCR) and ITS-2 (314 bp, sequencing) were obtained, a subset of 76 eggs was selected to describe ribosomal and mitochondrial haplotypes by full ITS1-5.8S-ITS2 (927 bp) and partial *cox*1 (long fragment, 1024 bp) sequencing (S1 Appendix). These 76 eggs included 35 lateral-spined eggs (*S. mansoni*-like), 29 collected from urine and six from stool, as well as 41 terminal-spined eggs (*S. haematobium*-like) collected from urine. The PCR reactions were performed in a final volume of 30 μL and comprising 8 μL of DNA template, as for the ITS-2. PCR amplifications for the ITSs region was performed with primers BD1 and BD2 [64], using an internal primer (4S) when necessary, following previously outlined PCR conditions [61,65,66]. For the *cox*1 gene, amplification was performed with the primers Cox1_schist F and Cox1_schist R [20], using PCR conditions as previously described [67].

PCR amplifications of ITS-2, 18S, ITS1-5.8S-ITS2, and *cox*1 (long fragment) were conducted using a Veriti 96-well thermal cycler (Applied Biosystems, Thermo Fisher Scientific, Waltham, MA, USA). PCR products (8–10 µL) from each of these four markers were visualized by electrophoresis on a 2% agarose gel stained with GelRed (50 min at 100V). The products were then purified using the Ultra Clean PCR Clean-up DNA Purification System (MoBio, Solana Beach, CA, USA) following the manufacturer’s protocol and resuspended in 50 μl of 10 mM TE buffer (pH 7.6). Final DNA concentration (in µg/ml) and the absorbance at 260/280 nm were determined using an Eppendorf BioPhotometer (Eppendorf, Hamburg, Germany).

Each DNA marker (ITS-2, 18S, ITS1-5.8S-ITS2, and *cox*1 long fragment) was independently PCR-amplified for each egg, and the resulting PCR products were sequenced on both strands using the dideoxy chain-termination method. Sequencing was conducted with the BigDye Terminator v3.1 Cycle Sequencing Kit (Applied Biosystems) on an Applied Biosystems 3730xl DNA Analyzer (Applied Biosystems, Foster City, CA, USA), using the same PCR amplification primers (5 µM) in accordance with the manufacturer’s protocol.

#### ITS-2 cloning and sequencing.

ITS-2 is a suitable marker for detecting recent hybridization, as nuclear ribosomal DNA can retain both parental copies for several generations before being homogenized by concerted evolution. However, the high divergence between *S. mansoni* and *S. haematobium* complicates the interpretation of sequences obtained through Sanger sequencing in cases of hybridization. To confirm that the heterozygous ITS-2 sequences observed in some *S. mansoni*-like eggs indeed represented copies from these two distinct parental species, we opted to clone a subset of 13 eggs (three from feces and 10 from urine) (S1 Appendix) that exhibited double peaks at multiple positions in their electropherograms, as has been done in similar situations [42]. The purified ITS-2 amplification products - using the previously mentioned commercial kit, the Ultra Clean PCR Clean-up DNA Purification System - from those 13 eggs, along with two controls of pure *S. haematobium* (genomic DNA, Valencia [61]) and pure *S. mansoni* (laboratory culture, Perpignan), were cloned.

Ligation was performed using the pGEM-T Easy Vector System I kit (Promega, Madison, WI, USA). Reaction for a final volume of 10 μL, included 5 μL 2X Rapid Ligation Buffer, 1 μl pGEM.-T Easy Vector (50 ng), 3 μL PCR product, and 1μL T4 DNA Ligase (3 Weiss units/μl). Reactions were incubated overnight at 4 ºC. Two μL of ligation reaction were transferred to a sterile 1.5 mL tube where 50 μL of *Escherichia coli* DH5α competent cells was added following the manufacturer instructions (https://www.promega.es/products/pcr/pcr-cloning/pgem-t-easy-vectorsystems/?catNum=A1360#protocols).

100 μl of each transformation culture were plated on LB/Ampicillin/IPTG/X-Gal medium. Following an overnight incubation period at 37°C, between six and eight distinct white colonies were PCR amplified and sequenced individually for each cloned sample. A freeze-thaw step (−20 °C, 10 min) was performed to lyse the bacteria prior to PCR amplification. The amplification and sequencing were conducted in accordance with the previously described conditions for ITS-2.

#### Sequence analysis.

Forward and reverse sequences of all rDNA and mtDNA markers were edited and assembled into a single corrected sequence for each individual DNA region with the software Sequencher v. 5.4.6 (Gene Codes Co. MI, USA). The alignment of these sequences was performed using default parameters with ClustalW in MEGA X software [68].

In the case of both the ITS-2 sequences and the complete intergenic region, ITS1-5.8S-ITS2, all nucleotide positions were carefully checked in the raw sequence chromatograms allowing for the detection of sequence polymorphisms between *S. haematobium* and *S. mansoni* (or *S. haematobium* and *S. bovis/S. curassoni*) and to look for possible heterozygosity, and differences in the height of the double peaks, especially in polymorphic positions that differentiate between these species, as previously described [22,24,69] (S2 Appendix). For the 18S sequences the five single nucleotide polymorphisms (SNPs) known to discriminate between *S. haematobium (Sh), S. bovis (Sb),* and *S. curassoni (Sc*) [70] were also meticulously reviewed. The reference sequences from GenBank used were: *Sh* from Mali and Tanzania (Z11976 and OX103963); *Sb* from Kenia (OX104095) and *Sc* from Senegal (AY157236) for the 18S gene; and *Sh* from Tanzania (OX103963), *Sb* from Kenia (OX104095) and *Sc* from Senegal (MT580946) for the ITSs.

The determination of ITS1-5.8S-ITS2 and *cox*1 (long fragment) haplotypes was carried out using the ALTER web server [71]. The establishment of homologies of each of the rDNA and mtDNA markers sequenced here was carried out using the BLAST program from the National Centre for Biotechnology Information website (http://www.ncbi.nlm.nih.gov/BLAST). Complete or almost complete sequences (with similar length in bp of the same molecular markers used in this study, 100% coverage and greater than 99% similarity in BLAST with our sequences) were retrieved from GenBank for sequence analysis comparisons, and haplotype identification. S1 (rDNA) and S2 Tables (mtDNA) included those retrieved sequences grouped by haplotypes/isolates to facilitate comparison analyses. Accession numbers were obtained after submission to the GenBank using BankIt submission tool of the National Center for Biotechnology Information (NCBI) (https://www.ncbi.nlm.nih.gov/genbank/) (Bethesda, Maryland, USA).

### Hybrid identification

#### Mito-nuclear code nomenclature.

The nomenclature utilized is the same used in previous studies [24,72], assuming that the *cox*1 gene is a mitochondrial marker of haploid inheritance, so that only one allele is indicated by the abbreviation of the species involved. The ITS-2 is a marker of biparental inheritance, so that each allele is indicated by the two-letter abbreviation of the identified species. For instance, the mito-nuclear signature for “pure” *S. mansoni* will be *S. mansoni cox*1 x *S. mansoni* ITS-2 = *SmxSmSm*.

Using this mito-nuclear code, we define hybrid eggs when the *cox*1 and ITS-2 markers are considered together and the results are species discordant and/or when heterozygous positions are detected in the ITS-2 sequences at points that discriminate between species (e.g., *S. mansoni cox*1 x *S. mansoni* x *S. haematobium* ITS-2 = *SmxSmSh*).

In the case of *S. haematobium, S. bovis*, and *S. curassoni*, the ITS-2 allows differentiation between *S. haematobium* and *S. bovis* or *S. curassoni* (*Sb/Sc*), but not between *S. bovis* and *S. curassoni*. Therefore, when detecting *S. haematobium cox*1 x *S. haematobium* x *S. bovis/S. curassoni* ITS-2, the 18S marker was used for species confirmation. Only in the case where there was no sufficient DNA for the sequencing of the 18S, the code *ShxShSb/Sc* was applied.

When no discordance is observed in the mito-nuclear signature, eggs are considered pure.

#### Haplotype code nomenclature.

The haplotype nomenclature used is organized by (i) identifying the species with a two-letter abbreviation or the species involved in the case of hybrids (as in the case of the mito-nuclear signature), (ii) the genetic marker used (ITSs, or *cox*1), and (iii) the haplotype (H) number (1, 2, 3, etc.). Heterozygotic haplotypes of rDNA hybrids are indicated by Htz. Examples: a pure *S. mansoni* haplotype from the complete intergenic region sequencing: Sm-ITSs-H1; a hybrid *S. mansoni x S. haematobium* haplotype: SmxSh-ITSs-Htz1; a hybrid *S. haematobium x S. curassoni* haplotype: ShxSc-ITSs-Htz1; a pure *S. mansoni* haplotype from partial sequencing of *cox*1: Sm-*cox*1-H1.

### Phylogenetic analyses

The phylogenetic analysis was performed using all *S. mansoni* and *S. haematobium cox*1 haplotypes identified in this study (11), together with 30 reference haplotypes obtained from GenBank (accession numbers are shown in the tree in the results section). The data matrix comprised 41 sequences. Following alignment, the sequences were standardized by trimming the 3’ or 5’ ends, as needed, to ensure congruence across at least 1024 positions in the final dataset. *Schistosoma japonicum* (KU196417) was used as outgroup. The best substitution model selection analysis was performed in MEGA X, taking into account the BIC (Bayesian Information Criterion) scores, the AICc (Akaike Information Criterion, corrected) value, the Maximum Likelihood (lnL) value, and the number of parameters (including branch lengths) for each model. The Maximun Likelihood (ML) method was used to infer the evolutionary history. To assess the reliability of the nodes in the trees, a bootstrap analysis using 1,000 replicates was made using the Bootstrap method in MEGA X.

## Results

### Mito-nuclear signatures

Of the 134 eggs examined, the complete mito-nuclear signature (*cox*1 + ITS-2) was obtained for 127 of them, of which 119 from urine and eight from stools (Table 2).

**Table 2:** Genetic profiles obtained according to their mito-nuclear signature, and organized by migrant country of origin, egg shedding morphology and type of sample processed (urine or stool).

	MITO-nuclear signature						
	*SmxSmSm*	*SmxSmSh*	*SmxSmSm*	*SmxSmSh*	*ShxShSh*	*ShxShSc*	*ShxShSb/Sc* ^*^
Genetic profile	*S. mansoni*	*S. mansoni* x *S*. *haematobium*	*S. mansoni*	*S. mansoni* x *S. haematobium*	*S. haematobium*	*S. haematobium* x *S. curassoni*	*S. haematobium* x *S. bovis/S. curassoni*
Classification	Pure	Hybrid	Pure	Hybrid	Pure	Hybrid	Hybrid
Egg morphology	*Sm*-like (urine)		*Sm*-like (stool)		*Sh*-like (urine)		
Country (patient code):							
Senegal (1Se)	3	–	6	**2**	7	–	–
Senegal (2Se)	–	**16**	–	–	23	–	**–**
Senegal (3Se)	–	**8**	–	–	9	–	–
Guinea-Bissau (1Gb)	–	**2**	–	–	21	–	**1** ^*^
Côte d’Ivoire (1 Ci)	–	**1**	–	–	8	**4**	–
Mali (1Ma)	–	**8**	–	–	8	**–**	–
Total	3	35	6	2	76	4	1

* Sample with ambiguous mito-nuclear signature, due to lack of DNA for 1369 bp. 18S sequencing.

In bold = eggs in which double peaks were found in the chromatogram of their ITS-2 sequences at polymorphic positions that discriminate between *S. mansoni* and *S. haematobium* or between *S. haematobium* and *S. bovis* or *S. curassoni*.

#### *S. mansoni-*like eggs.

All the 46 lateral-spined eggs (*S. mansoni-*like), 38 from urine + eight from stools, showed an *S. mansoni cox*1 profile (375 bp) by RD-PCR. Regarding ITS-2 sequences, only nine eggs (9/46, 19.57%) provided an ITS-2 corresponding to pure *S. mansoni*, while in 37 eggs (37/46, 80.43%) double chromatogram peaks were found at the ITS-2 polymorphic positions that discriminate between *S. mansoni* and *S. haematobium*. When the two markers were considered, two mito-nuclear signatures were obtained: (i) the pure *S. mansoni* genetic profile *SmxSmSm* was detected in only in three eggs (6.52%) from urine and in six eggs (13.04%) from stool, all from a single Senegalese patient (patient No. 1Se in Table 1); (ii) the hybrid genetic profile *SmxSmSh* was obtained in 37 eggs shed mainly by urine (35/37, 94.59%) and less in stools (2/37, 5.41%) of patients from Senegal, Guinea-Bissau, Côte d’Ivoire, and Mali (Table 2).

#### *S. haematobium*-like eggs.

All the 81 terminal-spined eggs (S*. haematobium*-like) furnished a *S. haematobium cox*1 profile by RD-PCR. None of the eggs provided a *S. mansoni* profile nor a *S. bovis/S.curassoni* profile*,* because RD-PCR cannot differentiate between *S. bovis* and *S. curassoni* [23,25]. According to ITS-2 sequences, 76 eggs (76/81, 93.83%) yielded an ITS-2 corresponding to pure *S. haematobium*, while in five eggs (5/81, 6.17%) double chromatogram peaks were found at the polymorphic positions that discriminate between *S. haematobium* and *S. bovis* or *S. curassoni*. Three mito-nuclear signatures were obtained when combining the results of the two markers: (i) *ShxShSh* (76/81, 93.83%) corresponding to a pure *S. haematobium* found in urine samples from Senegal, Guinea-Bissau, Côte d’Ivoire and Mali; and (ii) two hybrid profiles including: *ShxShSc* (4/81, 4.94%) of a 18S-sequencing-confirmed hybrid between *S. haematobium* and *S. curassoni* in the patient form Côte d’Ivoire; and a *ShxShSb/Sc* hybrid (1.23%) in the Guinea-Bissau patient, because of the impossibility of species identification due to insufficient DNA for 18S sequencing (Table 2).

It is worth mentioning that the patients from Côte d’Ivoire and Guinea-Bissau simultaneously presented two different hybrid mito-nuclear signatures: *SmxSmSh* in lateral-spined eggs from urine, and *ShxShSc* (in patient 1 Ci) or the undetermined *ShxShSb/Sc* (in patient 1Gb) in terminal-spined eggs from urine (Table 2).

### ITS-2 sequences obtained by cloning

The presence of both *S. mansoni* and *S. haematobium* ITS-2 sequences in each of the 13 (*S. mansoni*-like) cloned eggs was confirmed by sequencing the 99 clones obtained. It is noteworthy that, among the 86 clonal sequences, 54.65% (47/86) correspond to a pure ITS-2 of *S. mansoni* and 43.02% (37/86) to a pure *S. haematobium*, identical to the GenBank sequences AY446082 and MG554667 of *S. mansoni* and *S. haematobium*, respectively.

No intraindividual sequence variation was detected among the clone sequences obtained from each single egg. However, an unexpected result was obtained in only two clone sequences (2/86, 2.33%) from two lateral-spined eggs of Senegalese origin. These clone sequences presented a single ITS-2 without double peaks, surprisingly including a mixed sequence with one species in the first part (*S. mansoni* or *S. haematobium*) followed by the sequence of the other species (*S. haematobium* or *S. mansoni*) in the remaining part (Table 3).

**Table 3:** ITS-2 sequences identified in the 86 clonal sequences derived from 13 *S. mansoni*-like eggs.

Sequence identification	Sequence code	Number of clones (n= 86)	Polymorphic positions ITS-2																									
			1 7	2 6	5 8	6 1	6 7	9 0	9 1	1 0 8	1 2 7	1 4 5	1 9 5	2 0 5	2 0 6	2 0 8	2 2 3	2 3 2	2 3 3	2 5 2	2 5 3	2 5 8	2 6 5	2 7 9	2 8 9	2 9 2	3 0 2	3 0 4
*S. haematobium*	Cl-Sh	37	**G**	**G**	**G**	**T**	**C**	**G**	**A**	**G**	**C**	**C**	**G**	**A**	**G**	**A**	**C**	**G**	**G**	**A**	**T**	**C**	**C**	**G**	**A**	**C**	**G**	**A**
*S. mansoni*	Cl-Sm	47	**A**	**A**	**T**	**A**	**T**	**A**	**G**	**A**	**T**	**T**	**A**	**T**	**A**	**T**	**T**	**A**	**A**	**–**	**–**	**T**	**T**	**T**	**T**	**T**	**A**	**T**
*Sm/Sh*	Cl-*SmSh*	1	**A**	**A**	**T**	**A**	**T**	**A**	**G**	**A**	**T**	**C**	**G**	**A**	**G**	**A**	**C**	**G**	**G**	**A**	**T**	**C**	**C**	**G**	**A**	**C**	**G**	**A**
*Sh/Sm*	Cl-*ShSm*	1	**G**	**G**	**G**	**T**	**C**	**G**	**A**	**G**	**C**	**C**	**A**	**T**	**A**	**T**	**T**	**A**	**A**	**–**	**–**	**T**	**T**	**T**	**T**	**T**	**A**	**T**

The numbers, to be read vertically, indicate the variable positions from the alignment performed with MEGA X, differentiating between *S. mansoni* and *S. haematobium*. Color in nucleotide positions: blue = nucleotides characterizing *S. haematobium*; orange = nucleotides characterizing *S. mansoni*.

The positive controls of *S. mansoni* and *S. haematobium* showed a pure *S. mansoni* or a pure *S. haematobium* profile, respectively, in 100% of their clone sequences obtained.

### rDNA ITS1-5.8S-ITS2 and mtDNA *cox*1 sequence analyses and haplotype identification

#### *S. mansoni*-like eggs.

**rDNA haplotypes:** The complete sequence of the nuclear rDNA intergenic region of the 35 subsampled lateral-spined eggs, collected from the urine and stool, was obtained. These 35 sequences once aligned provided 3 different haplotypes: two pure *S. mansoni* and one hybrid *S. mansoni* x *S. haematobium* (Table 4).

**Table 4:** Molecular haplotype identification by rDNA (ITS1-5.8S-ITS2) and mtDNA *cox*1 sequencing of lateral and terminal spined eggs, according to the type of sample (urine or stool), patient, and country of origin. Codes for patients listed in Table 1.

	Sample	No of times obtained	rDNA + mtDNA haplotypes		Pure/hybrid classification	Country of origin	Patients
			ITSs	*cox*1			
Lateral-spined eggs (n = 35)	**Urine**						
		3	Sm-ITSs-H2	Sm-*cox*1-H21	Pure Sm	Senegal	1Se
		1	SmxSh-ITSs-Htz1	Undet^*^	Hybrid SmxSh	Guinea-Bissau	1Gb
		1	SmxSh-ITSs-Htz1	Sm-*cox*1-H23	Hybrid SmxSh	Côte d’Ivoire	1 Ci
		17	SmxSh-ITSs-Htz1	Sm-*cox*1-H24	Hybrid SmxSh	Senegal	2Se, 3Se
		2	SmxSh-ITSs-Htz1	Sm-*cox*1-H2	Hybrid SmxSh	Senegal	3Se
		4	SmxSh-ITSs-Htz1	Sm-*cox*1-H9	Hybrid SmxSh	Mali	1Ma
		1	SmxSh-ITSs-Htz1	Sm-*cox*1-H25	Hybrid SmxSh	Mali	1Ma
	**Stool**						
		1	SmxSh-ITSs-Htz1	Sm-cox1-H23	Hybrid SmxSh	Senegal	1Se
		2	Sm-ITSs-H3	Sm-*cox*1-H21	Pure Sm	Senegal	1Se
		1	Sm-ITSs-H2	Sm-*cox*1-H22	Pure Sm	Senegal	1Se
		2	Sm-ITSs-H3	Sm-*cox*1-H23	Pure Sm	Senegal	1Se
Terminal-spined eggs (n = 41)	**Urine**						
		6	Sh-ITSs-H3	Sh-*cox*1-H1	Pure Sh	Guinea-Bissau, Senegal, Mali	1Gb, 1Ma
		23	Sh-ITSs-H4	Sh-*cox*1-H1	Pure Sh	Senegal, Côte d’Ivoire	1Se, 2Se, 3Se, 1 Ci
		3	Sh-ITSs-H4	Sh-*cox*1-H9	Pure Sh	Guinea-Bissau	1Gb
		1	Sh-ITSs-H4	Sh-*cox*1-H18	Pure Sh	Côte d’Ivoire	1 Ci
		1	Sh-ITSs-H5	Sh-*cox*1-H1	Pure Sh	Côte d’Ivoire	1 Ci
		2	Sh-ITSs-H5	Sh-*cox*1-H35	Pure Sh	Mali	1Ma
		1	ShxSb/Sc-ITSs-Htz1	Undet^*^	Hybrid ShxSb/Sc	Guinea-Bissau	1Gb
		2	ShxSc-ITSs-Htz1	Sh-*cox*1-H1	Hybrid ShxSc	Côte d’Ivoire	1 Ci
		2	ShxSc-ITSs-Htz2	Sh-*cox*1-H1	Hybrid ShxSc	Côte d’Ivoire	1 Ci

* Sample not haplotyped due to lack of DNA for 1024 bp. *cox*1 sequencing.

**Fig 2:**
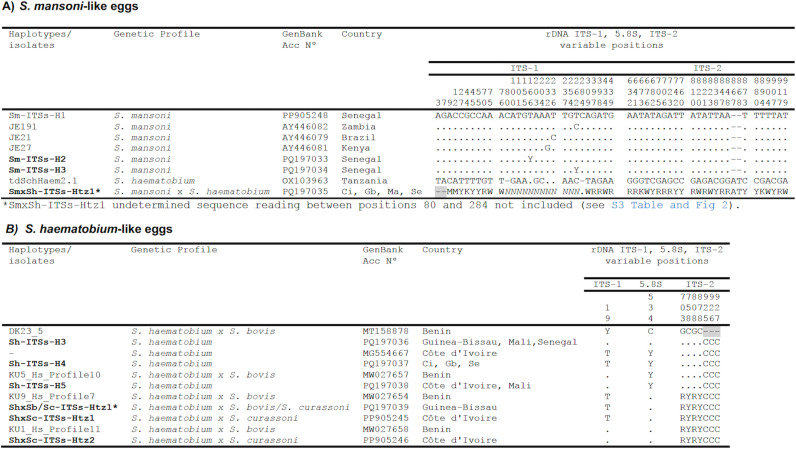
Polymorphic sites in the sequence comparison of the complete transcribed spacer region of the nuclear rDNA between the haplotypes of A) *S. mansoni* like-eggs and B) *S. haematobium* like-eggs haplotypes (H) obtained (in bold) and other haplotypes or isolates from GenBank (S1 Table). Numbers (to be read in vertical) refer to variable positions in the alignment made with MEGA X;. = Identical; - = Indel; - = Not sequenced; **N** = Undetermined nucleotide base; Heterozygotic position/s represented with corresponding symbol of IUPAC code for incomplete nucleic acid specification. Ci = Côte d’Ivoire; Gb = Guinea-Bissau; Ma=Mali; Se = Senegal.

The two pure haplotypes (Sm-ITSs-H2 and Sm-ITSs-H3) yielded a 927-bp-long intergenic region sequence differing at two heterozygotic positions located in the ITS-1 (at positions 166 and 262 of their alignment). These two haplotypes are “pure” *S. mansoni,* since the heterozygotic positions are not species discriminating but only evidence of intraspecific variability (Fig 2A).

**Fig 3:**
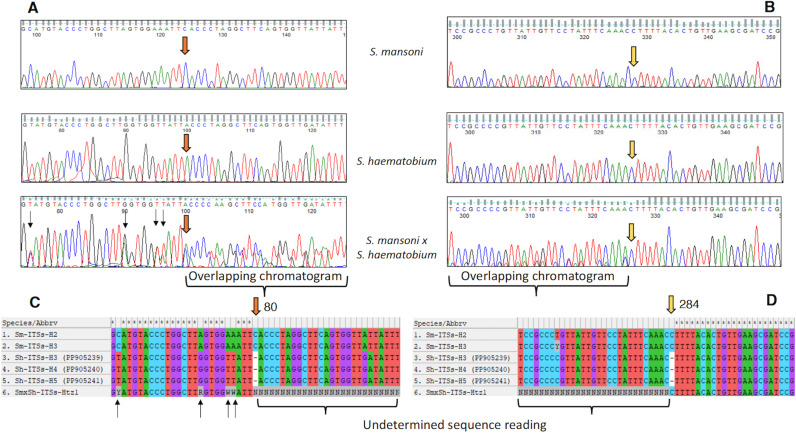
Two different fragments of ITS chromatograms showing the onset of the overlapping reading with the A) forward and B) reverse primers, respectively, in*S. mansoni x S. haematobium* sequences. The overlapping chromatograms reading with both primers is caused by two deletions in the *S. haematobium* sequence or two insertions in the *S. mansoni* sequence at positions 80 (brown arrow) (**C**) and 284 (yellow arrow) (**D**) of their alignment. This does not allow the nucleotide assignment from positions 80 to 283 of the 5’ ITS-1 region in eggs containing an *S. mansoni x S. haematobium* ITSs sequence. Black arrows indicate heterozygous positions at sites that discriminate between *S. mansoni* and *S. haematobium*.

The 927-bp-long intergenic region of the hybrid *S. mansoni* x *S. haematobium* haplotype (SmxSh-Htz1) showed a sequence with double peaks (being identical or very similar in heigh) in the chromatograms (Fig 3 and S2 Appendix), at positions of the ITS-1 and ITS-2 that differentiate *S. mansoni* from *S. haematobium*. In the ITS-1 sequence of this heterozygotic haplotype, nucleotide assignment from positions 80 to 284 was impossible due to the non-matching overlap of the two sequences (*S. mansoni and S. haematobium*). This impossibility is generated by a nucleotide absence in positions 80 and 284 of the *S. haematobium* sequence which correlate with the existence of two nucleotides in these positions of the *S. mansoni* sequence when aligning their forward and reverse sequences (Fig 3 and S3 Table).

**mtDNA haplotypes:** *Cox*1 sequencing provided seven 1024-bp-long *S. mansoni* mtDNA haplotypes (Sm-*cox*1-H2, Sm-*cox*1-H9, H21-H25) whose alignment show 19 intraspecific variable positions, of which, 7 parsimony-informative (p-info) and 12 singleton sites. Among the seven *Sm* haplotypes, the five including Sm-*cox*1-H2, plus H21 to H24, are detected in Senegal. This country shared Sm-*cox*1-H23 with Côte d’Ivoire. These seven haplotypes are for the first time reported for Senegal, Mali and Côte d’Ivoire.

**Fig 4:**
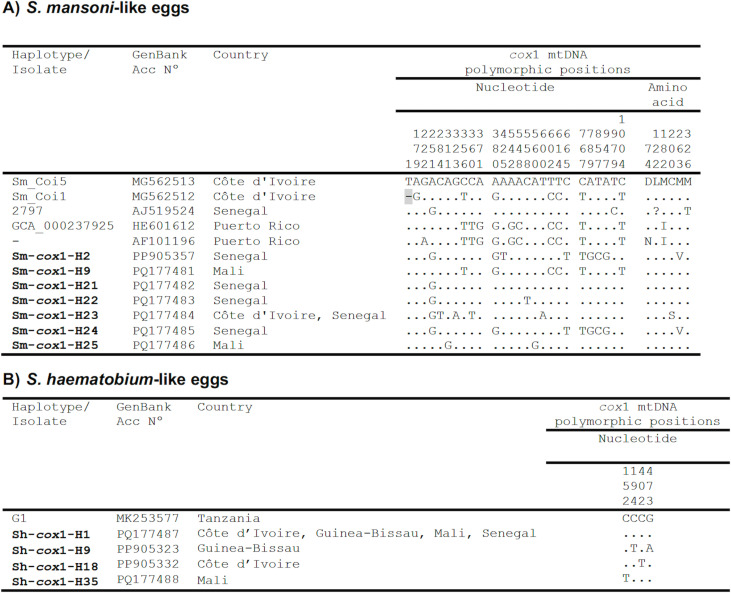
Polymorphic sites in nucleotide and amino acid positions identified in the *cox*1 mtDNA sequence alignment of *S. mansoni*-like and *S. haematobium*-like haplotypes **(H)** (in bold) and isolates of the same species from GenBank. Numbers (to be read in vertical) refer to variable positions obtained in the alignment made with MEGA X.= Identical; - = Not sequenced;? = undetermined positions. In the case of identical sequences (S2 Table), only one GenBank accession number has been selected as representative.

The comparison by alignment of the corresponding 340-aa-long COXI protein sequences show only three amino acid differences. Nucleotide and amino acid differences with other isolates from GenBank are listed in Fig 4A.

#### *S. haematobium*-like eggs.

**rDNA haplotypes:** The complete intergenic nuclear rDNA region sequence of the 41 *S. haematobium-*like eggs provided six haplotypes. Three of them correspond to a “pure” of *S. haematobium* profile and three to a hybrid profile *S. haematobium* x *S. bovis or S. curassoni.* The three 927-bp-long “pure” *S. haematobium* haplotypes Sh-ITSs-H3, H4 and H5 only differ at the two unexpected polymorphic positions 19 and 534 of their ITS1-5.8S-ITS2 alignment. These positions do not discriminate between *Schistosoma* species, rather, they represent intraspecific variability (Fig 2B).

The haplotype Sh-ITSs-H4, previously reported in Côte d’Ivoire (MG554667), Benin (MT158876, MW027655) and Corsica (MW130296), proved to be the most abundant in all our patients from Senegal, Guinea-Bissau and Côte d’Ivoire. The Sh-ITSs-H3 haplotype found in three patients from Senegal, Côte d’Ivoire, and Mali, and the Sh-ITSs-H5 haplotype found in the patients from Côte d’Ivoire and Mali proved to be identical to two *S. haematobium* haplotypes previously described in schoolchildren from Benin (MT158878 and MW027657, respectively) (Fig 2B and S1 Table).

The 18S sequencing of the three hybrid haplotypes allowed to assign two from Côte d’Ivoire as *Sh x Sc* (ShxSc-ITSs-Htz1 and Htz2). The third from Guinea-Bissau remained as ShxSb/Sc-ITSs-Htz1, because no sufficient DNA was available for 18S sequencing. The two hybrids *Sh x Sc* provided the two different 927-bp-long haploypes ShxSc-ITSs-Htz1 and ShxSc-ITSs-Htz2, which are characterized by presenting four heterozygous positions in ITS-2 and a differing mutation in ITS-1. These haplotypes have been previously reported in Beninese schoolchildren (MT158877, MW027654, and MT158880, MW027658) (Fig 2B and S1 Table).

**mtDNA haplotypes:** All terminal-spined eggs showed a 1024-bp-long *cox*1 sequence including four different *S. haematobium* haplotypes: Sh-*cox*1-H1, Sh-*cox*1-H9, Sh-*cox*1-H18 and Sh-*cox*1-H35. The fourth *S. haematobium* haplotypes showed 4 polymorphic sites in their alignment, which were all singleton sites. The 340-aa-long *COX*1 protein was identical for all nucleotide haplotypes. The most frequent haplotype Sh-*cox*1-H1 was found in the four countries and was identical to other haplotypes/isolates previously reported in: human samples from Tanzania (MK253577) and the Democratic Republic of the Congo (KY967520); in the snail *B. globosus* in Malawi (EU567128); and in the laboratory host (*Mesocricetus auratus*) from Gabon (KT354659-60). The haplotypes Sh-*cox*1-H9, Sh-*cox*1-H18 and Sh-*cox*1-H35 are for the first time reported for Senegal, Mali, Guinea Bissau and Côte d’Ivoire (Fig 4B and S2 Table).

### Individual egg characterization by rDNA and mtDNA haplotyping

The characterization of each egg was made by together considering the ribosomal (ITS1-5.8S-ITS2) and the mitochondrial (*cox*1) haplotypes obtained. This genetic characterization has allowed us to describe 17 different combinations of haplotypes in the total of eggs analyzed, among which nine for *S. mansoni*-like eggs and eight for *S. haematobium*-like eggs (Table 4).

As expected, the SmxSh-ITSs-Htz1 + Sm-*cox*1-H24 was the most frequently detected in the 17 *Sm*-like eggs (17/35, 48.57%) from the urine of two Senegalese patients. The Sh-ITSs-H4 + Sh-*cox*1-H1 was the most common in 23 *Sh*-like eggs (23/41, 50.09%) from the urine of three Senegalese patients and one Ivorian patient. Among the 17 combinations of rDNA and mtDNA haplotypes, only two were shared between countries and 15 are exclusive to each country analyzed. It is worth noting that we have detected up to six different combinations of haplotypes within the same patient, as found in the Ivorian patient. All the six patients studied presented simultaneously hybrid and pure haplotypes involving two or three *Schistosoma* species (Tables 2 and 4).

### *Cox*1 phylogenies

**Fig 5:**
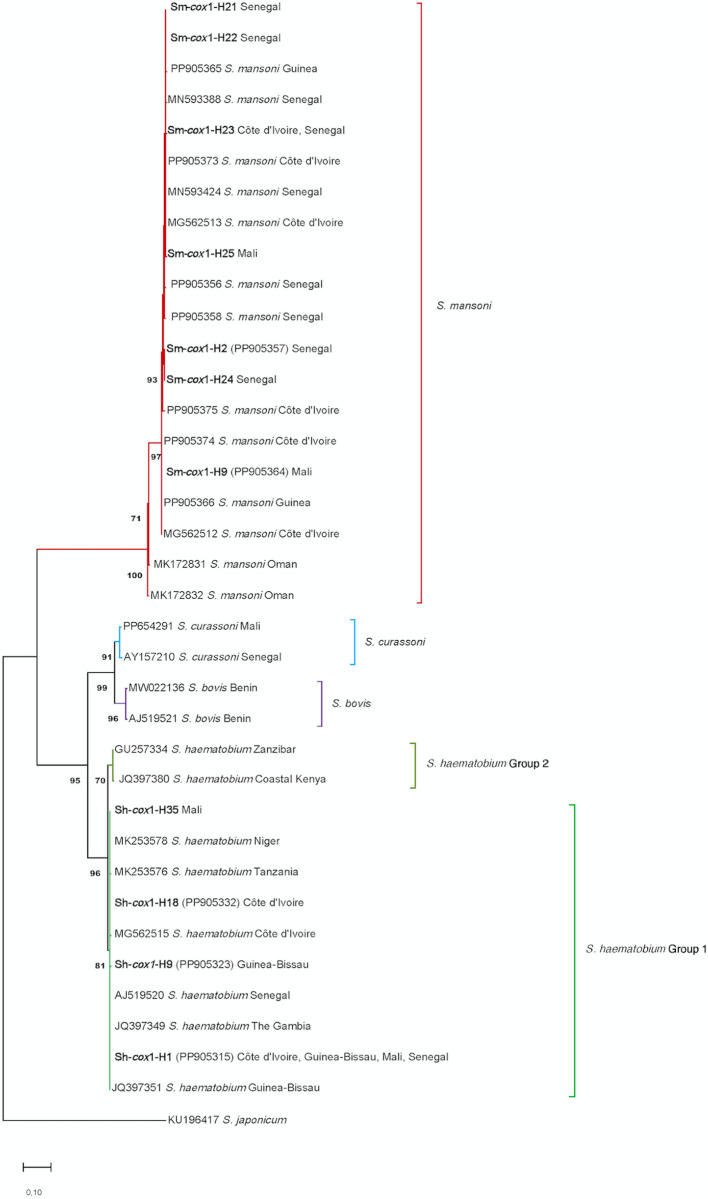
Phylogenetic tree of mtDNA *cox*1 of *Schistosoma* species based on maximum-likelihood model constructed with 11 haplotypes (7 *Schistosoma mansoni* and 4 *S. haematobium*) from the present study (in bold) and 30 sequences from the Genbank database (accession numbers are shown in the tree). Tree rooted using the *S. japonicum* sequence (KU196417) as outgroup. The tree is drawn to scale, with branch lengths measured as the number of substitutions per site. Bootstrap supports for nodes obtained using 1,000 replicates.

The Hasegawa-Kishino-Yano model with discrete gamma distribution (HKY+G) was the ML model that best fit our *cox*1 data set. The resulting ML tree (log likelihood = −3886.28) was inferred with five categories + Gamma parameter of 0.2016, and the rate variation model allowed for some sites to be evolutionarily invariant (Fig 5). This analysis involved 41 nucleotide sequences, including our *S. mansoni* and *S. haematobium cox*1 haplotypes and other *Schistosoma* spp. isolates or haplotypes from GenBank and *S. japonicum* (KU196417) as outgroup. There was a total of 1024 positions in the final dataset.

The seven *S. mansoni cox*1 haplotypes (Sm-*cox*1-H2, Sm-*cox*1-H9, Sm-*cox*1-H21-H25) obtained from lateral-spined eggs from urine and stool of migrant patients from Senegal, Côte d’Ivoire and Mali clustered in a well-supported monophyletic clade (100%) with other representative haplotypes of *S. mansoni*. Within this monophyletic clade, our seven haplotypes grouped in a well-supported branch (97%) with other *S. mansoni* haplotypes from Côte d’Ivoire, Guinea and Senegal (Fig 5).

The four *S. haematobium cox*1 haplotypes (Sh-*cox*1-H1, Sh-*cox*1-H9, Sh-*cox*1-H18 and Sh-*cox*1-H35) obtained from terminal-spined eggs from the urine appear grouped with other representative haplotypes of *S. haematobium* with a high support too (95%) (Fig 5). All four *S. haematobium cox*1 haplotypes obtained in this study appear inside the so-called group 1, which includes parasites from mainland Africa [73].

## Discussion

### Genetic characterization of *Schistosoma* eggs shed ectopically

Several studies have reported the presence of ectopic lateral-spined eggs (*S. mansoni-*like eggs) in human urine [34,39,43,45,46], but very few have characterized them genetically. In each of the only two studies that focused on the genetic characterization of these ectopic *S. mansoni*-like eggs, genotyping concerned only a low number of eggs from a single patient [45,46]. In our study, a total of 127 eggs were successfully genotyped using a combination of RD-PCR, nuclear rDNA and mtDNA marker sequencing, plus cloning. These 127 eggs genetically analyzed to identify their genetic profiles and molecular haplotypes, included: (i) 38 ectopic lateral-spined eggs (*S. mansoni-*like eggs) from the urine of six migrant patients residing in Spain and original from the four different countries of Côte d’Ivoire, Guinea-Bissau, Mali and Senegal); (ii) eight eggs from the stools of one of the Senegal patients; and (iii) 81 terminal-spined eggs (*S. haematobium-*like eggs) that were simultaneously shed in their urine.

The presence of ectopic lateral-spined eggs in urine can be explained through sexual interaction between *S. mansoni* and *S. haematobium*, or due to a “spilling-over” caused by high *S. mansoni* infection loads [43]. All six patients reported in this study showed *S. mansoni x S. haematobium* hybrid eggs, demonstrating a heterospecific crossbreeding between these two species. These results indicate that *S. haematobium* males may have mated with *S. mansoni* females in the bladder veins, leading to the production of lateral-spined eggs in urine. In the case of simple co-infection, as previously described in one Senegalese patient, no ectopic eggs or any hybrid eggs were detected [57]. It should be highlighted that of the total number of patients diagnosed with schistosomiasis at the TMU Almería, Spain (April 2018 - July 2024), 24.49% (12/49) were infected with *S. mansoni*, and 50.00% (6/12) among them proved to be carriers of *Sm x Sh* hybrid eggs.

Although unexpected *S. mansoni* and *S. haematobium* interactions have been observed in sporadic cases of Senegalese and Ivorian children [1], our results lead us to believe that the heterospecific crosses between these two species and the resulting hybrids may be common in areas where they are co-endemic and, as already suggested [46], probably more widespread than previously observed (in the anamnesis, only the patient from Guinea-Bissau mentioned to have taken a bath in a river of Senegal). This should be highlighted when considering the number of mixed *S. mansoni* and *S. haematobium* infection studies conducted in different African countries reporting ectopic shedding of *Schistosoma* eggs [34,39,43] without providing a genetic characterization of the eggs.

### Genetic heterospecific interactions between *S. mansoni* x *S. haematobium*

The fact that we have detected *S. mansoni x S. haematobium* hybrid eggs in only lateral-spined *S. mansoni*-like eggs, is in line with previous observations in two Ivorian children diagnosed in French hospitals [45,46]. All hybrids identified in lateral-spined eggs from urine showed a *SmxSmSh* mito-nuclear signature (Table 2). These results suggest that *S. haematobium* males have mated and migrated with *S. mansoni* females to the veins surrounding the bladder, resulting in the production of lateral-spined eggs in urine, which seems consistent with the competitiveness and the higher proportion of *S. haematobium* adult males compared to *S. mansoni* [43,74].

We have also detected *S*. *mansoni* x *S. haematobium* hybrid eggs in the stool of the only patient from Senegal (see 1Se in Table 1). Both in northern Senegal [42] and in one of the aforementioned Ivorian children [46], hybrids were detected in stool. While the study conducted in Senegal does not refer to egg morphology, the French study alludes to *S. mansoni* morphology, which is in concordance with our results. Traditionally, it has always been assumed that it is up to the male to determine the location of oviposition due to its critical role in carrying the female [75], while egg morphology has been associated with the female, as it is in the ootype that the shape is determined [74,76,77].

The presence of a hybrid egg with a lateral spine in the feces could also be explained by a possible migration error or the influence of the hybrid nature of the parasite on the migration of the pair disrupting the usual tropism of parasites, as demonstrated in *S. bovis x S. haematobium* hybrids [78]. Although we cannot totally exclude the possibility that stool samples were contaminated with urine during collection, it would be interesting to conduct further studies on mating behavior and cross-breeding experiments between these two species with clearly visually different eggs to better understand the inheritance of egg morphology and the possibilities and limitations of the resulting hybrids. In that sense, it should be highlighted that hybrid schistosome eggs may exhibit different morphotype variability [67].

Bidirectional inheritance has been described in *S. mansoni x S. haematobium* hybrids, since some eggs/miracidia with a mixed ITSs profile (*S. mansoni x S. haematobium*) had a *S. mansoni cox*1 haplotype, while others displayed a *S. haematobium* haplotype [42,46]. However, we did not identify a *S. haematobium cox*1 profile among genotyped hybrid lateral spined eggs. A fecal *SmxSmSh* egg as the result of a second-generation hybrid (F1 male *SmxSmSh* crossing with female *SmxSmSh* or pure *Sm* female) would imply that *SmxSh* F1 progeny could infect a molluscan host, which is so far still not evident [79]. The production of *S. mansoni x S. haematobium* hybrids is still quite surprising since they belong to two different evolutionary lineages of schistosomes. According to their phylogeny, it was traditionally suspected that their pairings would result in predominantly parthenogenetic egg production and, if viable, would only be able to infect the molluscan host spectrum of the maternal schistosome species, as recently demonstrated experimentally between *S. mansoni* and *S. bovis* [79]. Although *S. bovis* and *S. haematobium* are closely related species, both belonging to the monophyletic *S. haematobium* clade, and a large unidirectional introgression of *S. bovis* into *S. haematobium* has been demonstrated [80], they do not interact equally with *S. mansoni*.

On one hand, *S. mansoni* and *S. bovis* can share the same rodent host, have the same tropism for the oviposition site, and their combination shows mate choice recognition, which represent a behavioral isolation associated with species belonging to two different evolutionary lineages [21]. On the other hand, *S. mansoni* and *S. haematobium*, which can also share the same host but different tropism inside the vertebrate host, can easily mate without preference [74] which is associated with closely related species interaction. It appears that pre-zygotic reproductive isolating traits have not been strongly selected for in *S. mansoni* and *S. haematobium* as they exist in *S. mansoni* and *S. bovis*, and, despite both being crosses between phylogenetically distant species, the former combination results in hybrid progeny and the latter in parthenogenetic offspring [79].

The presence of *Schistosoma* hybrids between human and animal species of the *S. haematobium* group, such as *S. haematobium* x *S. bovis* or *S. haematobium* x *S. curassoni*, has been widely reported in Senegal [22], Côte d’Ivoire [24], Cameroon [81] and Mali [25,82], including recently in a migrant from Guinea-Bissau [72]. However, hybrids resulting from the interaction of the two major human species, *S. mansoni* x *S. haematobium*, have only been genetically confirmed in Senegal and Côte d’Ivoire [42,45,46]. In Guinea-Bissau and Mali, these hybrids have never been reported before. Although it is impossible to verify that patient 1Gb (see Tables 1 and 2) was infected in his country of origin, since he confirmed bathing in the Senegal River, our study suggests that hitherto unknown hybridization areas may exist.

### Emergence risk of *SmxSh* schistosome infection in Europe

The viability of the resulting *S. mansoni x S. haematobium* progeny has not yet been evaluated. In the study conducted in Senegal [42], the authors were unable to infer anything about the viability of the hybrid offspring. They found no intra-individual variation in cloned ITSs copies and a high number of *S. haematobium* copies compared to *S. mansoni* clone copies within the two free-living miracidia they analyzed. These results may indicate that these two miracidia concerned subsequent generations or backcross hybrids, although more data are needed to confirm this observation. In our study, more *S. mansoni* copies than *S. haematobium* were obtained within the 86 clone sequences from the 13 lateral-spined eggs cloned from Guinea-Bissau, Senegal, Côte d’Ivoire and Mali. Given the minimal intra-individual variation that we detected in cloned copies of ITS-2, it is possible that *S. mansoni x S. haematobium* lateral-spined eggs are first or early generation hybrids.

Although we detected mobile miracidia inside the eggs and some even hatched after manipulation under the microscope (Fig 1), we did not analyze the potential viability of these eggs. Experimental studies are definitely needed to assess the viability and transmission of the offspring resulting from crosses between *S. mansoni* and *S. haematobium*, which would help understanding their potential impact on the epidemiology of the disease. In non-endemic areas, schistosomiasis introduction may occur when a competent intermediate host snail is locally available [83]. In the case of Europe, we have clear examples of schistosomiasis transmission or potential transmission due to the presence of snail vectors, as the case of *B. truncatus* in Corsica or Spain and *B. tenagophila* in Romania, where this susceptible snail species was not only introduced, but established [51]. Given the lower stringency with which schistosomatids select intermediate hosts [84,85], as well as their ability to infect other mollusk groups such as lymnaeids [86], the potential for host switching and invasion of new areas warrants attention, even though transmission has not yet been confirmed.

Further research is required to assess whether *S. mansoni* x *S. haematobium* hybrids can infect the *Bulinus* snail vector species commonly found across European countries as France, Spain, Italy, Greece, and Portugal [52,54,87].

### Hybrid and pure infections according to the patient and country of origin

To our knowledge, this is the first time to report six patients shedding urinary ectopic lateral-spined eggs simultaneously presenting two types of hybrid mito-nuclear signatures involving three *Schistosoma* species: *SmxSmSh* (in *S. mansoni*-like eggs), and *ShxShSc* or *ShxShSb/Sc* (in *S. haematobium*-like eggs,) as in patients from Guinea-Bissau and Côte d’Ivoire. These results highlight the importance of the appropriate molecular identification of the genetic variants that are being introduced not only in Spain, but also in Europe. Our study reports on hybrids between distant species (*Sm x Sh*), but also on hybrids between species of the same group (*Sh x Sc* or *Sh* x *Sb/Sc*), as well as pure species (*Sm* or *Sh*) that clinically cause two different types of pathology, such as intestinal schistosomiasis and genitourinary schistosomiasis.

The potential impact that S*. mansoni x S. haematobium* hybrids (*SmxSmSh*), and two types of hybrids (*SmxSmSh* and *ShxShSc* or *SmxSmSh* and *ShxShSb/Sc*) involving three *Schistosoma* species (*S. mansoni, S. haematobium* and *S. curassoni/S. bovis*), on patient’s morbidity and treatment response should be taken into account. Recent studies on migrant patients in Spain from West African countries who shed *Schistosoma* hybrid eggs appear to experience higher morbidity. However, this does not seem to affect the results of diagnostic tests or the clinical and analytical responses. Hybrid infections apparently lead to a greater number of genitourinary lesions and severe complications of the disease may increase in the long-term, such as hydronephrosis or bladder cancer [57]. In a study conducted in the endemic region of northern Senegal, an increase in hepatic morbidity but a decrease in urogenital morbidity was observed, along with reduced improvement following treatment with praziquantel (PZQ), in patients infected with zoonotic hybrids compared to non-hybrids [88].

A study, using the mtDNA *cox*1 and the rDNA intergenic region, demonstrated an interaction between three species of the same *S. haematobium* group (*S.b* x *S.h* x *S.c*) in the Tillabéri region, Niger [89]. From the epidemiological point of view, *S. haematobium* and the *S. haematobium* group hybrids appeared to be more specific and were only transmitted by *B. truncatus* [89], the same snail species present in the Almeria region where our six patients lived and where autochthonous transmission has been reported [19].

The six patients reported here are from countries where schistosomiasis is endemic: Senegal, Guinea-Bissau, Côte d’Ivoire, and Mali. Both *S. haematobium* and *S. mansoni* are known in Senegal [90–92], Côte d’Ivoire [24,93–95] and Mali [96,97]. In these countries, studies were already conducted on mixed infection prevalence [34,98], morbidity [37,99,100] and model-based estimation co-distribution [101]. However, knowledge on the epidemiology of schistosomiasis in Guinea-Bissau is scarce, with only one study on prevalence and morbidity due to *S. haematobium* infection [102], and absence of data regarding other *Schistosoma* species.

### Types of genetic signatures, hybrids and haplotypes detected

In the present study, the 127 successfully genotyped *Schistosoma* eggs allow us to describe five genetic signatures, two pure and three hybrids, involving *S. mansoni, S. haematobium, S. curassoni and S. bovis/S. curassoni* and 20 rDNA and mtDNA haplotypes. The majority of these haplotypes (13/20, 65.0%) are first findings in Spain-imported schistosomes and also new for the original African countries, especially the hybrid haplotype SmxSh-ITSs-Htz1.

This is the first time that a great number of ribosomal and mitochondrial haplotypes are described in patients with simultaneous lateral and terminal-spined eggs in urine. The ITSs and *cox*1 sequence results of each individualized egg revealed a total of 17 different combinations, including up to six different haplotypes detected within the same patient (Table 4).

## Conclusion

The results allow us to corroborate a high hybridization complexity (Tables 2 and 4):

- In the urine of each one of five patients from Senegal, Guinea-Bissau, Côte d’Ivoire and Mali, *S. mansoni x S. haematobium* hybrid eggs (in *Sm*-like eggs) and also pure *S. haematobium* eggs (*Sh*-like) were found. In a sixth patient from Senegal, *S. mansoni x S. haematobium* hybrid eggs (*Sm*-like) and also pure *S. mansoni* eggs (*Sm*-like) in feces, together with pure *S. haematobium* eggs (*Sh*-like) and *S. mansoni* eggs (*Sm*-like) in urine, were found. In the urine of the patient from Côte d’Ivoire, *S. haematobium x S. curassoni* hybrid eggs (*Sh*-like) were found in addition to *S. mansoni x S. haematobium* hybrid eggs (*Sm*-like) and pure *S. haematobium* eggs (*Sh*-like). In the urine of the patient from Guinea-Bissau, *S. mansoni x S. haematobium* hybrid eggs (*Sm*-like), pure *S. haematobium* eggs (*Sh*-like), and *S. haematobium x S. bovis/S. curassoni* hybrid eggs (*Sh-*like) were found.
- This illustrates (i) a high frequency of different hybrid types and (ii) the presence of up to three species involved in such hybrids within the urine of the same patient. *S. mansoni x S. haematobium* hybrid eggs were mostly detected in *S. mansoni*-like eggs from urine (94.59%), whereas in feces the detection of those hybrids was less frequent (5.41%).
- In the total of *S. mansoni*-like eggs analyzed (urine and stools), 80.44% were hybrids (*Sm x Sh*) and only 19.56% were pure *S. mansoni.* Within *S. haematobium*-like eggs (only in urine, no ectopic shedding), 93.83% were pure *S. haematobium*, while only a 6.17% were hybrids (*Sh x Sc* or *Sh x Sb/Sc*).
- The unexpected *Sm x Sh* hybridization appears to be frequent among the migrant population of the four countries studied, and it is the first time that it is reported for Guinea-Bissau and Mali.
- The three patients from Senegal provide the greatest variety of hybrids, but always within the *Sm x Sh* profile and always in eggs with *S. mansoni* morphology. In this country, pure *S. haematobium* and pure *S. mansoni* genetic profiles were also detected, in concordance with the egg morphology.

The detection and genetic characterization of these hybrids between *Schistosoma* species at a geographical distance demonstrates not only the frequency with which they are entering non-endemic countries, such as Spain and consequently in Europe, but also poses a wake-up call concerning the clinical presentations, diagnostic challenges, treatment responses and epidemiological impact of potential transmission and establishment in non-endemic areas. It is evident that further studies on the potential genetic crossbreeding giving rise to *Schistosoma* hybrids, as well as on their viability, are needed.

## Supporting information

S1 Table Description of the 86 nuclear ribosomal DNA sequences including the complete intergenic region (ITS1-5.8S-ITS2) from GenBank used for comparison purposes, according to their hosts and geographical origin. (PDF)

S2 Table Distribution of the 25 *cox*1 sequences from GenBank used for comparison purposes, according to their hosts and geographical origin. (PDF)

S3 Table Variable positions in the complete intergenic region (ITS1-5.8S-ITS2) alignment between pure *S. mansoni*, pure *S. haematobium* and hybrid *S. mansoni x S. haematobium* sequences obtained. (PDF)

S1 Appendix Primers used for RD-PCR and PCR amplification and sequencing and a summary of *S. mansoni* and *S. haematobium* like-eggs processed for DNA genotyping by RD-PCR, sequencing and cloning. (PDF)

S2 Appendix Some examples of RD-PCR agarose gels displaying the mitochondrial *cox*1 profiling, along with partial chromatograms of the ITS-2 sequences from some of the *Sm x Sh* hybrid eggs. (PDF)
