# Correction: *Schistosoma mansoni x S. haematobium* hybrids frequently infecting sub-Saharan migrants in southeastern Europe: Egg DNA genotyping assessed by RD-PCR, sequencing and cloning

**DOI:** 10.1371/journal.pntd.0013121

**Published:** 2025-05-20

**Authors:** The PLOS Neglected Tropical Diseases Staff

There are errors in the author affiliations. The correct affiliations are as follows:

Alejandra De Elías-Escribano^1,2^, Patricio Artigas^1,2^, Joaquín Salas-Coronas^2,3,4^, María Pilar Luzon-Garcia^2,3,4^, Marta Reguera-Gomez^1^, María Isabel Cabeza-Barrera^2,3,4^, José Vázquez-Villegas^2,3,5^, Jerôme Boissier^6^, Santiago Mas-Coma^1,2^, María Dolores Bargues^1,2^

**1** Departamento de Parasitología, Facultad de Farmacia, Universidad de Valencia, Burjassot, Valencia, Spain,

**2** Centro de Investigación Biomédica en Red de Enfermedades Infecciosas (CIBERINFEC), Instituto de Salud Carlos III, Madrid, Spain,

**3** Tropical Medicine Unit, Hospital Universitario Poniente, El Ejido, Almería, Spain,

**4** International Health Research Group of Almería (GISIA), Faculty of Health Sciences, University of Almería, La Cañada de San Urbano, Almería, Spain,

**5** Tropical Medicine Unit, Distrito Sanitario Poniente de Almería, El Ejido, Almería, Spain,

**6** IHPE, Univ. Montpellier, CNRS, IFREMER Université de Perpignan Via Domitia, Perpignan, France

The S3 Table is incorrect. Please view the correct S3 Table below.

The publisher apologizes for the errors.
