# Correction: Correction: *Schistosoma mansoni* x *S. haematobium* hybrids frequently infecting sub-Saharan migrants in southeastern Europe: Egg DNA genotyping assessed by RD-PCR, sequencing and cloning

**DOI:** 10.1371/journal.pntd.0013438

**Published:** 2025-08-19

**Authors:** The PLOS Neglected Tropical Diseases Staff

The S3 Table is omitted from the list of Supporting Information in the correction published on May 20, 2025. It can be viewed below. The publisher apologizes for the error.

## Supporting information

S3 Table Variable positions in the complete intergenic region (ITS-1, 5.8S, and ITS-2) alignment between pure *S. mansoni*, pure *S. haematobium* and hybrid *S. mansoni* x *S. haematobium* sequences obtained. (PDF)
