# Supplementary material for: Schistosoma mansoni x S. haematobium hybrids frequently infecting sub-Saharan migrants in southeastern Europe: Egg DNA genotyping assessed by RD-PCR, sequencing and cloning
Source: PLoS Negl Trop Dis. 2025 Mar 31;19(3):e0012942. doi: 10.1371/journal.pntd.0012942 (PMC11984978; doi:10.1371/journal.pntd.0012942)
Supplement: S1 Table — (PDF) [file pntd.0012942.s001.pdf]

S1 Table

Description of the 86 nuclear ribosomal DNA sequences including the complete intergenic region (ITS-1, 5.8S and ITS-2) from GenBank used for comparison purposes, according to their hosts and geographical origin: **A)** 27 sequences of *S. mansoni*, and the corresponding 4 haplotypes they provided. **B)** 59 sequences of *S. haematobium, S. curassoni, S. guineensis, S. bovis*, *S. haematobium x S. bovis* and the corresponding 21 haplotypes they provided**;** Bp = base pairs of the ITS rDNA region available in GenBank for each sample. Identical sequences of different haplotype /isolate are grouped and separated by horizontal lines.

A)

Haplotype/isolate	Organism	GenBank Acc.No.	Host	Locality	Country	Length (bp)
-	*S. mansoni*	AF531314	*Biomphalaria sudanica*	Lake Victoria	Tanzania	927
"SmITSLALV"	*S. mansoni*	FJ750523	*Homo sapiens*	Lake Albert región	Uganda	923
ITSa1	*S. mansoni*	JQ289742	-	-	Brazil	915
ITSa2	*S. mansoni*	JQ289743	-	-	Senegal	915
ITSa3	*S. mansoni*	JQ289744	-	-	Mali	915
ITSa4	*S. mansoni*	JQ289745	-	-	Nigeria	915
ITSa5	*S. mansoni*	JQ289746	-	-	Uganda	915
ITSa6	*S. mansoni*	JQ289747	-	-	Kenya	915
ITSa7	*S. mansoni*	JQ289748	-	-	Saudi Arabia	915
ITSa8	*S. mansoni*	JQ289749	-	-	Oman	915
ITSa9	*S. mansoni*	JQ289750	-	-	Egypt	915
"X2"	*S. mansoni*	MF776590	*Mastomys huberti*	Nder, Lac de Guiers	Senegal	903
JE191	*S. mansoni*	AY446082	*Homo sapiens*	Kariba Dam	Zambia	927
ITSb1	*S. mansoni*	JQ289751	-	-	Brazil	915
ITSb2	*S. mansoni*	JQ289752	-	-	Senegal	915
ITSb3	*S. mansoni*	JQ289753	-	-	Uganda	915
ITSb4	*S. mansoni*	JQ289754	-	-	Kenya	915
JE21	*S. mansoni*	AY446079	*Homo sapiens*	Sumidouro	Brazil	927
-	*S. mansoni*	KX011041	*Gorilla gorilla gorilla*	-	Gabon	955
-	*S. mansoni*	KX011042	*Pan troglodytes troglodytes*	-	Gabon	955
ITSc1	*S. mansoni*	JQ289755	-	-	Mali	915
ITSc2	*S. mansoni*	JQ289756	-	-	Kenya	915
-	*S. mansoni*	MG554659	-	-	Côte d'Ivoire	902
JE27	*S. mansoni*	AY446081	*Biompahalaria pfeifferi*	Makueni, Makindu Town	Kenya	927
ITSd1	*S. mansoni*	JQ289757	-	-	Senegal	915
ITSd2	*S. mansoni*	JQ289758	-	-	Nigeria	915
ITSd3	*S. mansoni*	JQ289759	-	-	Kenya	915

B)

Haplotype/isolate	Organism	GenBank Acc.No.	Host	Locality	Country	Length (bp)
tdSchHaem2.1	*S. haematobium*	OX103963	-	-	Tanzania	927
tdSchHaem1.1	*S. haematobium*	OX104046	-	-	Egypt	927
KE14_26	*S. haematobium*	MT158873	*Homo sapiens*	Kessounou	Benin	924
pSHTSA1- A2, pSHTSC1, pSHTSB3, pSHTSF3	*S. haematobium*	Z21716	clone	-	Mali	923
Mwenje_BGL_7	*S. haematobium*	MT884914	*Bulinus globosus*	-	Zimbabwe	927
DK23_5	*S. haematobium x S. bovis*	MT158878	*Homo sapiens*	Kessounou	Benin	924
-	*S. haematobium*	MG554667	-	-	Côte d'Ivoire	927
ITS_2019	*S. haematobium*	MW130296	-	Corsica	France	927
KU10_Hs_	*S. haematobium*	MW027655	*Homo sapiens*	Kessounou	Benin	927

Profile8	*x S. bovis*					
DK54_16	*S. haematobium x S. bovis*	MT158876	*Homo sapiens*	Kessounou	Benin	924
hyShITSBt	*S. haematobium x S. bovis*	FJ588857	*Bulinus truncatus*	Senegal River Basin	Senegal	926
hyShITSmir	*S. haematobium x S. bovis*	FJ588858	*Homo sapiens*	Senegal River Basin	Senegal	926
hyShITSBg	*S. haematobium x S. bovis*	FJ588859	*Bulinus globosus*	Senegal River Basin	Senegal	926
hymixShITSmir	*S. haematobium x S. bovis*	FJ588860	*Homo sapiens*	Senegal River Basin	Senegal	926
ShITSmir	*S. haematobium*	FJ588861	*Homo sapiens*	Senegal River Basin	Senegal	926
ShITSb	*S. haematobium*	JQ397401	-	-	Mali	926
-	*S. haematobium x S. bovis*	MK358844	*Homo sapiens*	Nsanje	Malawi	915
-	*S. haematobium*	MK358856	*Homo sapiens*	Mangochi	Malawi	915
-	*S. haematobium*	MK358857	*Homo sapiens*	Nsanje	Malawi	915
-	*S. haematobium*	MK358858	*Homo sapiens*	Chikhawa	Malawi	915
-	*S. haematobium*	MK797748	*Rattus rattus*	-	France	926
BK17_BB7.2	*S. haematobium*	MT580953	*Homo sapiens*	Barkedji	Senegal	926
BK17_LLod1.2	*S. haematobium*	MT580954	*Bulinus umbilicatus*	Loumbel Lode	Senegal	926
BK17_FH21.6	*S. haematobium x S. bovis*	MT580955	*Homo sapiens*	Loumbel Lana	Senegal	926
RT15_Ye1-10.6	*S. haematobium x S. bovis*	MT580957	*Bulinus truncatus*	Yetti-Yone, Lac de Guiers	Senegal	926
RT16_NW51.3	*S. haematobium*	MT580959	*Homo sapiens*	Richard Toll	Senegal	926
-	*S. haematobium*	GU257398		Zanzibar	Tanzania	926
KU5_Hs_Profile10	*S. haematobium x S. bovis*	MW027657	*Homo sapiens*	Kessounou	Benin	927
DK23_23	*S. haematobium x S. bovis*	MT158879	*Homo sapiens*	Kessounou	Benin	924
BK17_KG1.4	*S. curassoni*	MT580946	*Bulinus umbilicatus*	Kangaledji	Senegal	926
BK17_BKG7.7	*S. curassoni*	MT580947	*Capra hircus*	Barkedji	Senegal	926
BK16.1_ BB8liv.8	*S. bovis x S. curassoni*	MT580948	*Bos indicus*	Linguere	Senegal	926
RT17_MbG8.6	*S. curassoni*	MT580961	*Capra hircus*	Mbane, Lac de Guiers	Senegal	926
V20M2_Rr_ Profile1	*S. bovis*	MW027648	*Rattus rattus*	Vekky	Benin	927
tdSchBovi2.1	*S. bovis*	OX104095	-	-	Kenya	927
tdSchBovi1.1	*S. bovis*	OX103954	-	-	Spain	927
tdSchGui1.1	*S. guineensis*	OX103898	-	-	São Tomé and Principe	927
BK19_1	*S. bovis*	MT158872	Cows	Kessounou	Benin	924
SbITSBt	*S. bovis*	FJ588862	*Bulinus truncatus*	Upper Senegal River Basin	Senegal	926
BK16.3_3BB13st.1	*S. bovis*	MT580950	Cattle, goat and sheep	Linguere	Senegal	926
RT17_DB19.8	*S. bovis*	MT580958	*Bos indicus*	Didjiery	Senegal	926
V20M1_Rr_ Profile2	*S. bovis*	MW027649	*Rattus rattus*	Vekky	Benin	927
BK19_2	*S. haematobium x S. bovis*	MT158874	Cows	Kessounou	Benin	924
V20M8_Rr_Profile4	*S. bovis*	MW027651	*Rattus rattus*	Vekky	Benin	927
V132M1_Rr_Profile3	*S. bovis*	MW027650	*Rattus rattus*	Vekky	Benin	927
K14M2_Mn_Profile5	*S. haematobium x S. bovis*	MW027652	*Homo sapiens, M. natalensis*	Kessounou	Benin	927
BK24_4	*S. haematobium x S. bovis*	MT158881	*Homo sapiens,* Cows	Kessounou	Benin	924
BK24_1	*S. haematobium x S. bovis*	MT158875	Cows	Kessounou	Benin	924
K14M1_Mn_Profile6	*S. haematobium x S. bovis*	MW027653	*Homo sapiens*	Kessounou	Benin	926

BK16_LLA15.7	*S. haematobium x S. bovis*	MT580951	*Homo sapiens*	Loumbel Lana	Senegal	926
BK16_LL1.1	*S. haematobium x S. bovis*	MT580952	*Homo sapiens*	Loumbel Lana	Senegal	926
RT16_NG55.5	*S. haematobium x S. bovis*	MT580960	*Homo sapiens*	Richard Toll	Senegal	926
KU9_Hs_Profile7	*S. haematobium x S. bovis*	MW027654	*Homo sapiens*	Kessounou	Benin	927
DK23_21	*S. haematobium x S. bovis*	MT158877	*Homo sapiens*	Kessounou	Benin	924
KU1_Hs_Profile11	*S. haematobium x S. bovis*	MW027658	*Homo sapiens*	Kessounou	Benin	927
DK23_11	*S. haematobium x S. bovis*	MT158880	*Homo sapiens*	Kessounou	Benin	924
RT16.1_Th1.2	*S. haematobium x S. bovis*	MT580956	*Bulinus truncatus*	Thiago, Lac de Guiers	Senegal	926
KU3_Hs_Profile9	*S. haematobium x S. bovis*	MW027656	*Homo sapiens*	Kessounou	Benin	927
Kg2_1	*S. haematobium x S. bovis*	MT158882	*Homo sapiens,* Cows	Kessounou	Benin	924
