# Supplementary material for: Schistosoma mansoni x S. haematobium hybrids frequently infecting sub-Saharan migrants in southeastern Europe: Egg DNA genotyping assessed by RD-PCR, sequencing and cloning
Source: PLoS Negl Trop Dis. 2025 Mar 31;19(3):e0012942. doi: 10.1371/journal.pntd.0012942 (PMC11984978; doi:10.1371/journal.pntd.0012942)
Supplement: S2 Table — (PDF) [file pntd.0012942.s002.pdf]

S2 Table

Distribution of the 25 *cox*1 sequences from GenBank used for comparison purposes, according to their hosts and geographical origin: **A)** 6 sequences of *S. mansoni,* and the corresponding 5 haplotypes they provided; **B)** 19 sequences of *S. haematobium,* and the corresponding 9 haplotypes they provided. Bp = base pairs of the *cox*1 gene available in GenBank for each sample. Identical sequences of different haplotype/isolate are grouped and separated by horizontal lines.

A)

Haplotype/ isolate	Organism	GenBank Acc.No.	Host	Locality	Country	Length (bp)
2797	*S. mansoni*	AJ519524	-	-	Senegal	1224
Sm_Coi1	*S. mansoni*	MG562512	*Homo sapiens*	-	Côte d'Ivoire	1029
Sm_Coi5	*S. mansoni*	MG562513	*Homo sapiens*	-	Côte d'Ivoire	1029
GCA_000237925	*S. mansoni*	HE601612	-	-	Puerto Rico	1806
-	*S. mansoni*	NC_002545	-	-	-	1532
-	*S. mansoni*	AF101196	-	-	Puerto Rico	1876

B)

Haplotype/ isolate	Organism	GenBank Acc.No.	Host	Locality	Country	Length (bp)
G1	*S. haematobium*	MK253577	*Homo sapiens*	Mwera	Tanzania	1806
Pointe Noire	*S. haematobium*	KY967520	*Homo sapiens*	Pointe Noire	Republic of the Congo	1175
-	*S. haematobium*	KT354659	*Mesocricetus auratus*	Ekouk	Gabon	1175
-	*S. haematobium*	KT354660	*Mesocricetus auratus*	Melen	Gabon	1175
Malawi-LgHap2	*S. haematobium*	EU567128	*Bulinus globosus*	Lake Malawi-Likoma	Malawi	1164
G1	*S. haematobium*	MK253567	*Homo sapiens*	Daikaina Bell	Niger	1806
-	*S. haematobium*	KT354661	*Mesocricetus auratus*	Toho	Benin	1175
tdSchHaem1.1	*S. haematobium*	OX104052	-	-	Egypt	1806
-	*S. haematobium*	MW067222	*Mesocricetus auratus*	-	Egypt	1806
-	*S. haematobium*	MW067223	*Mesocricetus auratus*	-	Egypt	1806
-	*S. haematobium*	MW067224	*Mesocricetus auratus*	-	Egypt	1806
-	*S. haematobium*	MW067225	*Mesocricetus auratus*	-	Egypt	1806
-	*S. haematobium*	MW067226	*Mesocricetus auratus*	-	Egypt	1806
-	*S. haematobium*	MW067227	*Mesocricetus auratus*	-	Egypt	1806
Malawi-LgHap1	*S. haematobium*	EU567127	*Bulinus globosus*	Lake Malawi-Likoma	Malawi	1164
-	*S. haematobium*	NC_008074	*Mesocricetus auratus*	-	Mali	1541
3572	*S. haematobium*	AJ519520	-	Mbodiene	Senegal	1224
G1	*S. haematobium*	MK253576	*Homo sapiens*	Kitope	Tanzania	1806
G1	*S. haematobium*	MK253578	*Homo sapiens*	Karma	Niger	1806
