# Supplementary material for: Schistosoma mansoni x S. haematobium hybrids frequently infecting sub-Saharan migrants in southeastern Europe: Egg DNA genotyping assessed by RD-PCR, sequencing and cloning
Source: PLoS Negl Trop Dis. 2025 Mar 31;19(3):e0012942. doi: 10.1371/journal.pntd.0012942 (PMC11984978; doi:10.1371/journal.pntd.0012942)
Supplement: S1 Appendix — (PDF) [file pntd.0012942.s004.pdf]

S1 Appendix

Primers for RD-PCR

**Cox1 (short fragment) RD-PCR primers name**		**Sequence**
Universal Forward	Forward	TTTTTTGGTCATCCTGAGGTGTAT
S. bovis	Reverse	CACAGGATCAGACAAACGAGTACC
S. mansoni	Reverse	TGCAGATAAAGCCACCCCTGTG
S. haematobium	Reverse	TGATAATCAATGACCCTGCAATAA

Primers for PCR amplification and sequencing

**ITS2 primers name**		**Sequence**
3S	Forward	CTGAACGGTGGATCACTCGGCTCGTG
28S	Reverse	GGGATCCTGGTTAGTTTCTTTTCCTCCGC

**18S primers name**		**Sequence**
BUG1	Forward	CTGGTTGATCCTGCCAGTAGTAGT
BUG608	Reverse	AACCGCAACAACTTTAATATAC
Lim501	Forward	GGCCCCGTAATTGGAATGAGTA
Lim1369	Reverse	CACCACCCACCGAATCAAGAAA

**ITS1-5.5S-ITS2 primers name**		**Sequence**
BD1	Forward	CGTGTAACAAGGTTTCCGTA
BD2	Reverse	TATCGTTAAATTCAGCGGGT
4S	Reverse	TCTAGATGCGTTCGAARTGTCGATG

**Cox1 (long fragment) primers name**		**Sequence**
Cox1_schist F	Forward	TCTTTRGATCATAAGCG
Cox1_schist R	Reverse	TAATGCATMGGAAAAAAACA

S. mansoni and *S. haematobium* like-eggs processed for DNA genotyping by RD-PCR, sequencing and cloning.

	MITO-NUCLEAR SIGNATURE							CLONATION		HAPLOTYPE IDENTIFICATION					
Genetic markers	*cox*1 RD-PCR			ITS-2 sequencing (313 bp)			18S sequencing (1369 bp)	ITS-2 sequencing (313 bp)		ITS-1, 5.8S, ITS-2 sequencing (927 bp)			*cox*1 sequencing (1024bp)		
Type of sample	Urine		Stool	Urine		Stool	Urine	Urine	Stool	Urine		Stool	Urine		Stool
Egg morphology	Sm-like	Sh-like	Sm-like	Sm-like	Sh-like	Sm-like	Sh-like	Sm-like	Sm-like	Sm-like	Sh-like	Sm-like	Sm-like	Sh-like	Sm-like
Country (patient code):															
Senegal (1Se)	3	7	9	3	7	9	-	-	3	3	7	6	3	7	6
Senegal (2Se)	16	24	-	16	24	-	-	9	-	11	5	-	11	5	-
Senegal (3Se)	10	9	-	10	9	-	-	-	-	8	9	-	8	9	-
Guinea-Bissau (1Gb)	2	22	-	2	22	-	1	1	-	1	8	-	1	8	-
Côte d'Ivoire (1Ci)	2	13	-	2	13	-	4	-	-	1	9	-	1	9	-
Mali (1Ma)	8	9	-	8	9	-	-	-	-	5	3	-	5	3	-
Processed eggs	41	84	9	41	84	9	5	10	3	29	41	6	29	41	6
Total		**134**			**134**		**5**	**13**			**76**			**76**	
