# Supplementary material for: Schistosoma mansoni x S. haematobium hybrids frequently infecting sub-Saharan migrants in southeastern Europe: Egg DNA genotyping assessed by RD-PCR, sequencing and cloning
Source: PLoS Negl Trop Dis. 2025 Mar 31;19(3):e0012942. doi: 10.1371/journal.pntd.0012942 (PMC11984978; doi:10.1371/journal.pntd.0012942)
Supplement: S2 Appendix — (PDF) [file pntd.0012942.s005.pdf]

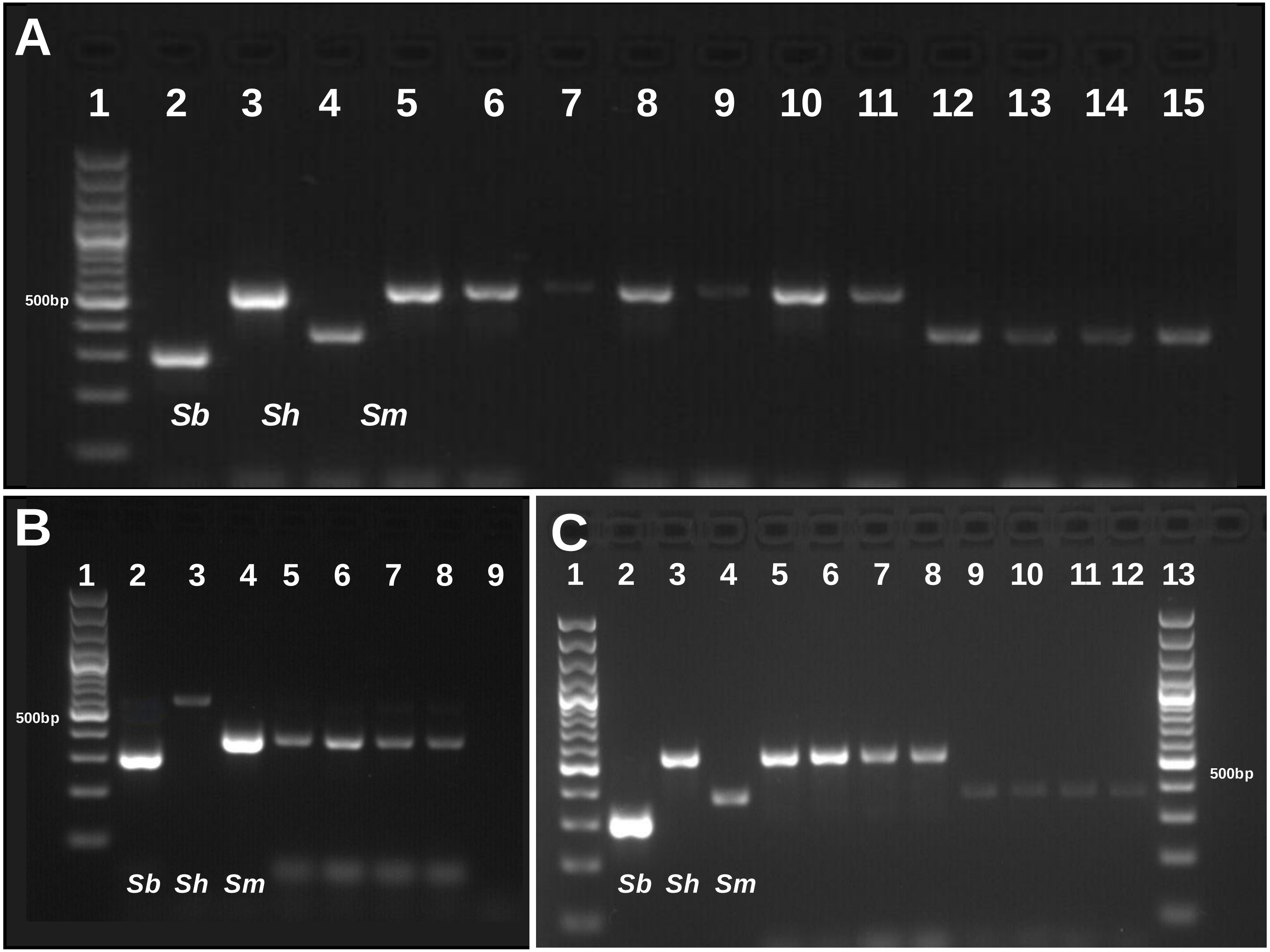
A
1 2 3 4 5 6 7 8 9 10 11 12 13 14 15
500bp
Sb Sh Sm
B
1 2 3 4 5 6 7 8 9
500bp
Sb Sh Sm
C
1 2 3 4 5 6 7 8 9 10 11 12 13
500bp
Sb Sh Sm

A

B

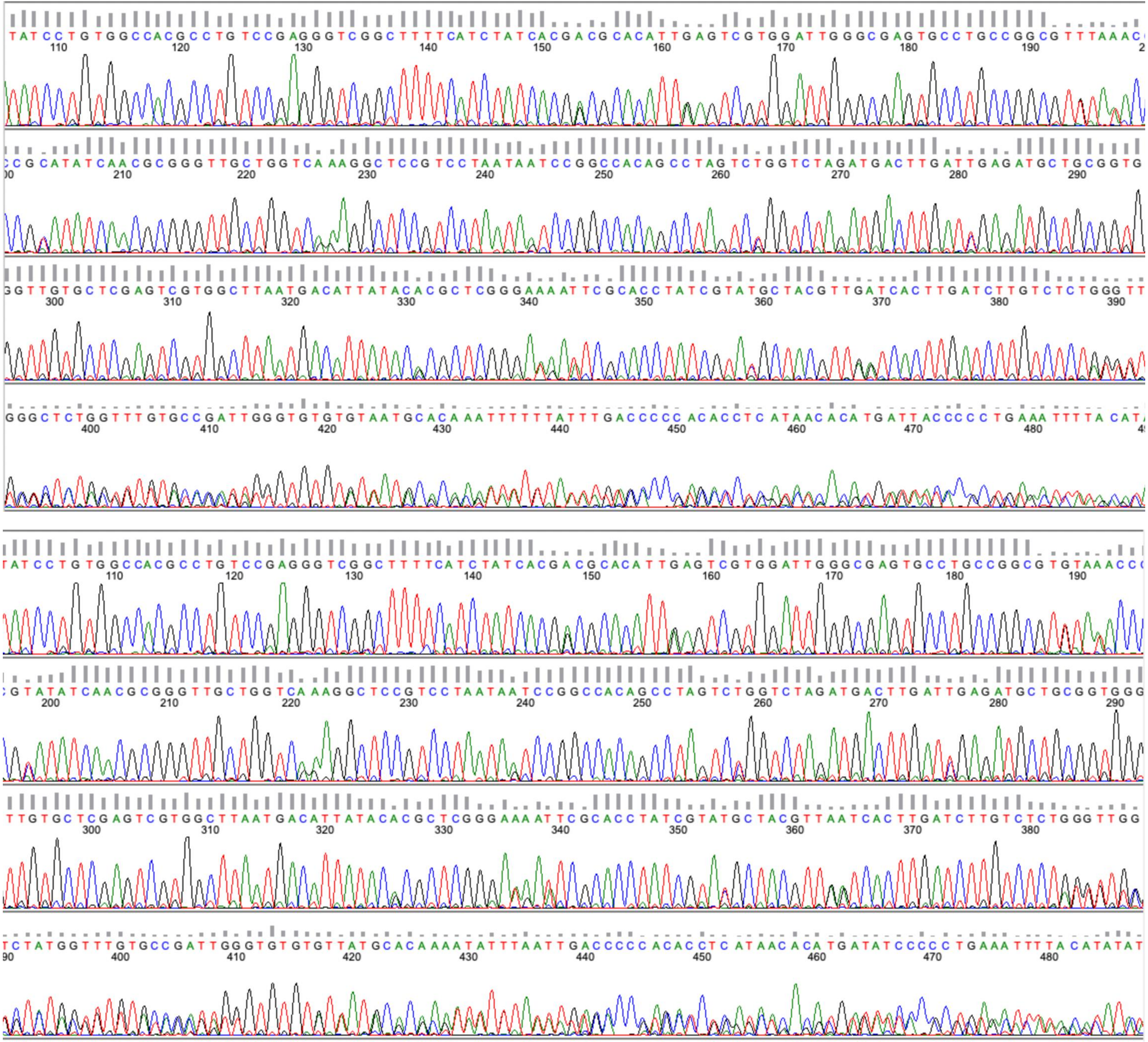

C

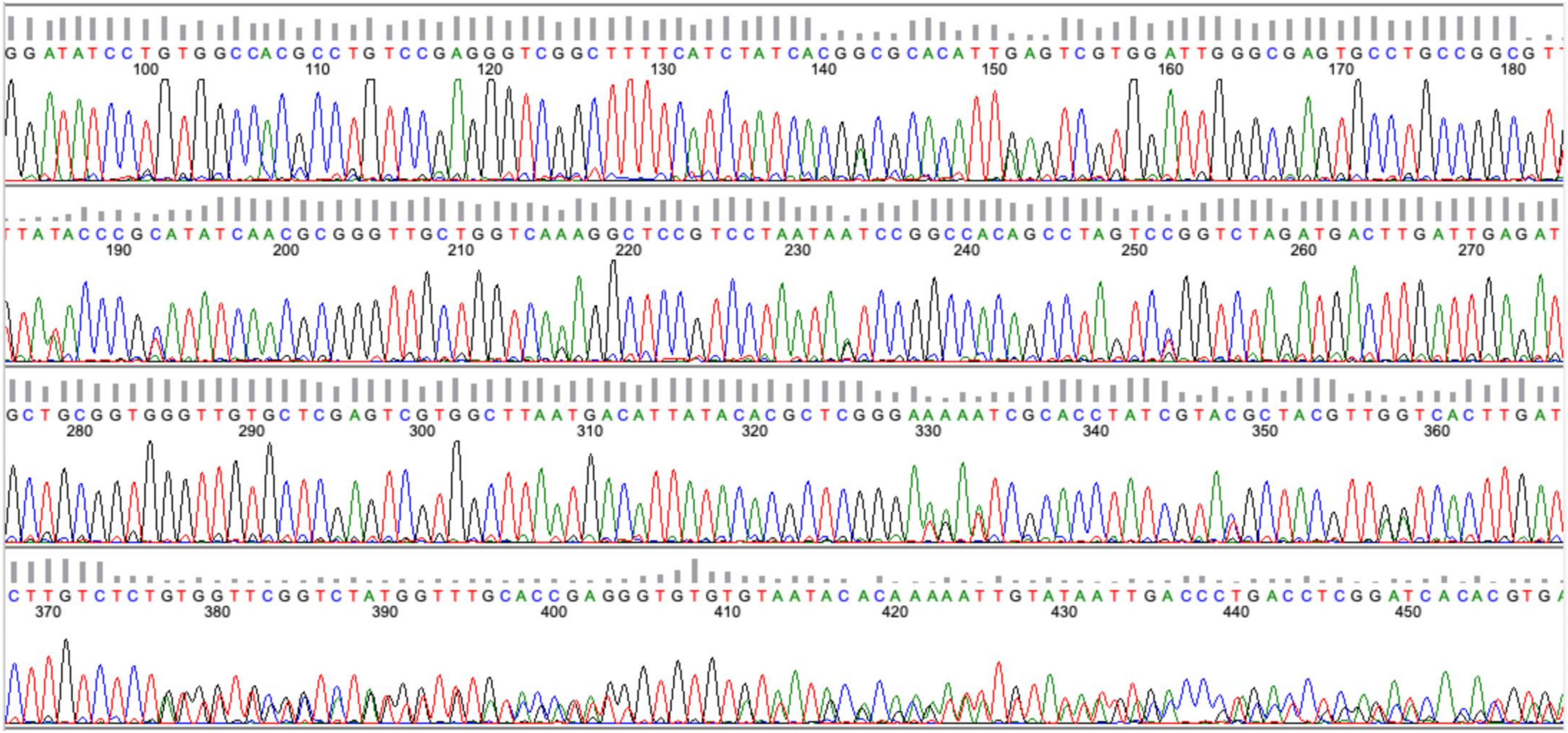

D

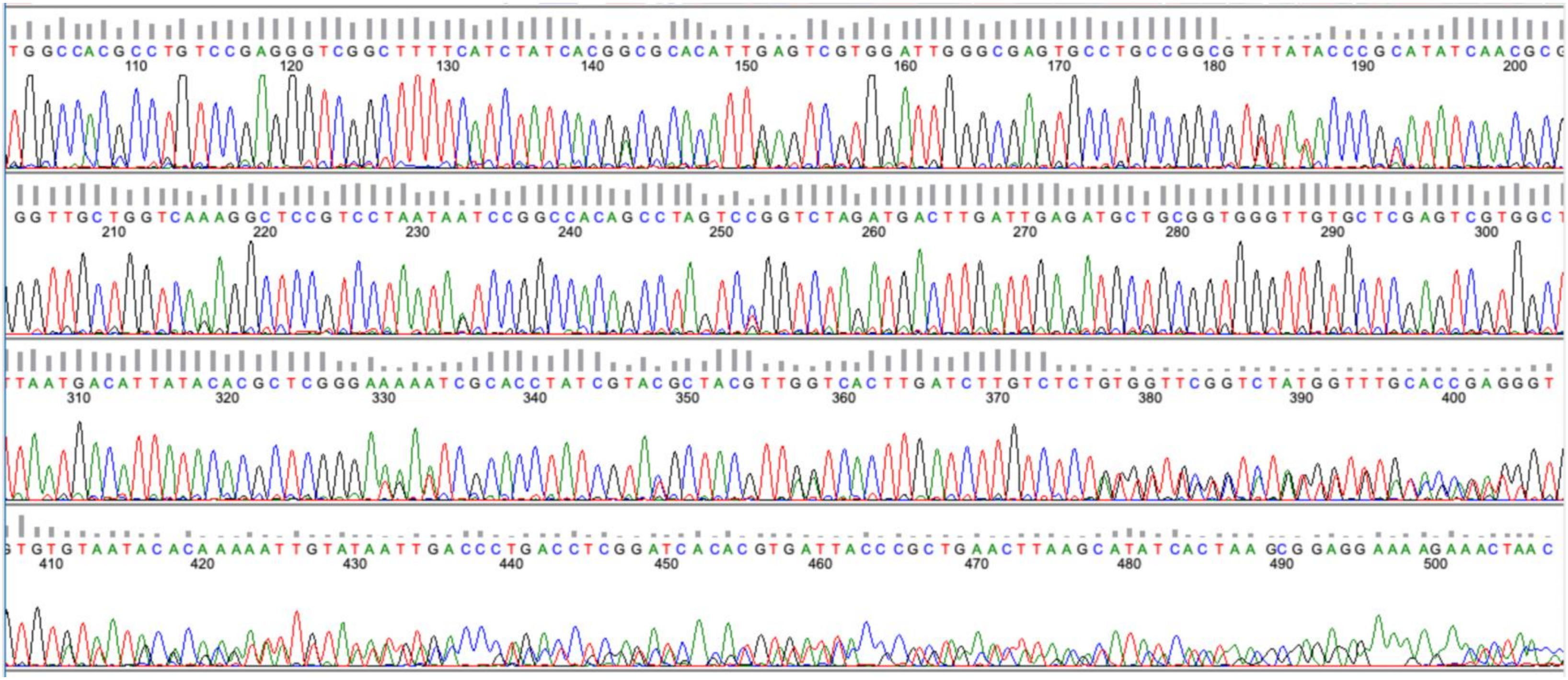

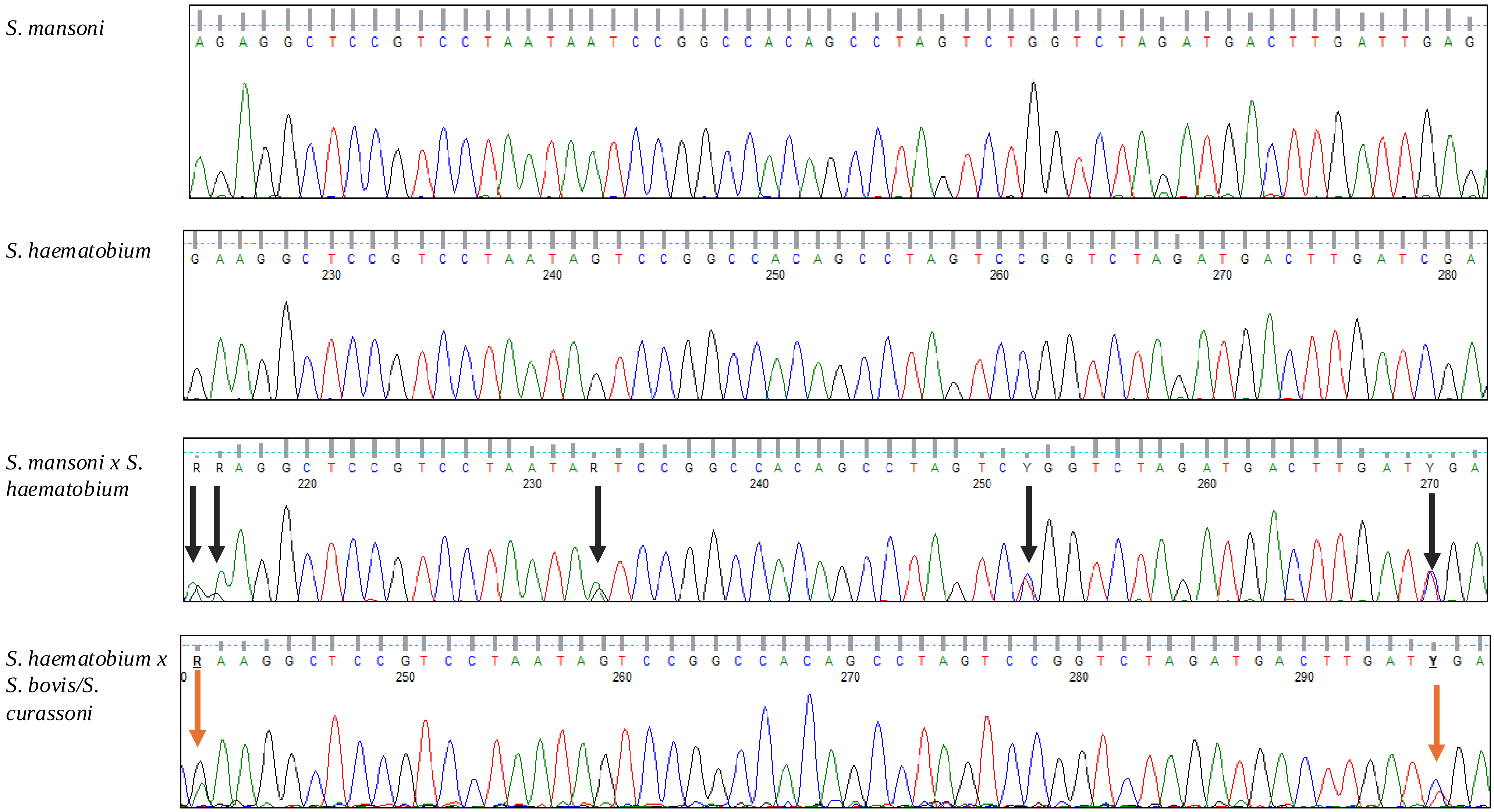
S. mansoni
S. haematobium
S. mansoni x S. haematobium
S. haematobium x S. bovis/S. curassoni

1.- Some examples of agarose gel showing the mitochondrial *cox*1 profiling by RD-PCR: A) Lane 1: DNA ladder (100-3000 bp); lane 2: *S. bovis* (306 bp); lane 3: *S. haematobium* (543bp); and lane 4: *S. mansoni* (375 bp). Each of the following lanes (5-15) contains RD_PCR products form each egg obtained from patient 2Se: lanes 5-11 are terminal-spined eggs (*S. haematobium*-like eggs) from urine; lanes 12-15 are lateral-spined eggs (*S. mansoni*-like eggs) from urine. B) Lane 1: DNA ladder (100-3000 bp); lane 2: *S. bovis* (306 bp); lane 3: *S. haematobium* (543bp); and lane 4: *S. mansoni* (375 bp). Each of the following lanes (5-9) contains RD_PCR products form each lateral-spined egg obtained from the stools of patient 1Se. C) Lane 1 and 13: DNA ladder (100-3000 bp); lane 2: *S. bovis* (306 bp); lane 3: *S. haematobium* (543bp); and lane 4: *S. mansoni* (375 bp). Each of the following lanes (5-12) contains RD_PCR products form each egg obtained from patient 1Ma: lanes 5-8 are terminal-spined eggs from urine; lanes 9-12 are lateral-spined eggs from urine.

2 - 3. - Some examples of partial row chromatograms of the ITS-2 forward sequence of hybrid *Sm x Sh* eggs: A) *S.mansoni*–like egg from the urine of the patient 3Se; B) *S.mansoni*-like egg from the urine of the patient 5Se; C) *S.mansoni*-like egg from the urine of the patient 4Ci; and D) *S.mansoni*-like egg from the urine the patient 2Se.

4.- Selected fragment of ITS-2 sequence chromatograms showing: a pure signal of *S. mansoni,* detected in lateral-spined eggs from urine and stool; a pure signal of *S. haematobium* detected in terminal-spined eggs from urine; a hybrid signal of *S. mansoni x S. haematobium* showing double peaks (marked with black arrows) at positions that discriminate between *S. mansoni* and *S. haematobium,* detected in lateral-spined eggs from urine and stool; and a hybrid signal of *S. haematobium x S. bovis/S. curassoni* presenting double peaks (marked with orange arrows) at positions that discriminate between *S. haematobium* and *S. bovis* or *S. curassoni,* detected in terminal-spined eggs from urine.
