# Supplementary material for: Correction: Correction: Schistosoma mansoni x S. haematobium hybrids frequently infecting sub-Saharan migrants in southeastern Europe: Egg DNA genotyping assessed by RD-PCR, sequencing and cloning
Source: PLoS Negl Trop Dis. 2025 Aug 19;19(8):e0013438. doi: 10.1371/journal.pntd.0013438 (PMC12364360; doi:10.1371/journal.pntd.0013438)
Supplement: S3 Table — (PDF) [file pntd.0013438.s001.pdf]

S3 Table

Variable positions in the complete intergenic region (ITS-1, 5.8S, and ITS-2) alignment between pure *S. mansoni*, pure *S. haematobium* and hybrid *S. mansoni x S. haematobium* sequences obtained. Numbers (to be read in vertical) refer to variable positions obtained in the alignment made with MEGA X; . = Identical; - = Indel; - = Not sequenced; N = Undetermined nucleotide base between positions 80 and 284 due to the considerable overlap of the two sequences (*S. mansoni and S. haematobium*); Heterozygotic position(s) represented by the corresponding symbol of the IUPAC code for incomplete nucleic acid specification. Ci = Côte d'Ivoire; Gb = Guinea-Bissau; Ma = Mali; Se = Senegal.

No.	Species/hybrid	GenBank Acc N°	Country	rDNA variable positions — ITS-1, 5.8S, ITS-2 — ITS-1
				111111111 1111111111 1111111111 1111111111 1111111111 1111111111
				1244577 7888888888 8999999999 9000000000 0111111111 1222222222 2333333333 3444444444 4555555555
				3792745505 6012345678 9012345678 9012345678 9012345678 9012345678 9012345678 9012345678 9012345678
1	*S. mansoni*	AF531314	Tanzania	AGACCGCCAA ACACCCTAGG CTTCAGTGGT TATTATTTTC TTGACCGGGG TACCTAGCCT GTCGTATGCC CTGATGGTGT TCTCGTGACT
2	*S. haematobium*	OX103963	Tanzania	TACATTTTGT T-........ .... .GA....... ....A...
3	*S. mansoni x S. haematobium*	-	Ci, Gb, Ma, Se	--MMYKYYRW W*NNNNNNNNN* *NNNNNNNNNN* *NNNNNNNNNN* *NNNNNNNNNN* *NNNNNNNNNN* *NNNNNNNNNN* *NNNNNNNNNN* *NNNNNNNNNN*

No.	rDNA variable positions (Continuation) — ITS-1, 5.8S, ITS-2 — ITS-1
	1111111111 1111111111 1111111111 1111111111 1222222222 2222222222 2222222222 2222222222 2222222222 2222222222 2222222222 2222222222 2222223334 4
	5666666666 6777777777 7888888888 8999999999 9000000000 0111111111 1222222222 2333333333 3444444444 4555555555 5666666666 6777777777 7888880993 3
	9012345678 9012345678 9012345678 9012345678 9012345678 9012345678 9012345678 9012345678 9012345678 9012345678 9012345678 9012345678 9012349784 9
1	TTCGGGTTGC CTGATCTGCC AAGGGCGATG GGACAGTGCA TGACAATATT GTTGTGTGCT AGGTTCAAAG AGAATTGTTT GCTATATGCA TGCAAGTCCG CCCTGTTATT GTTCCTATTT CAAACCAGAT G
2	....GC.... ....A. ....A.... ...C...... ....-TAGA A
3	*NNNNNNNNNN* *NNNNNNNNNN* *NNNNNNNNNN* *NNNNNNNNNN* *NNNNNNNNNN* *NNNNNNNNNN* *NNNNNNNNNN* *NNNNNNNNNN* *NNNNNNNNNN* *NNNNNNNNNN* *NNNNNNNNNN* *NNNNNNNNNN* *NNNNN*.WRRW R

No.	rDNA variable positions (Continuation) — ITS-1, 5.8S, ITS-2 — ITS-2
	6666677777 8888888888 889999
	3477800246 1222344667 890011
	2136256320 0013878783 044779
1	AATATAGATT ATATTAA--T TTTTAT
2	GGGTCGAGCC GAGACGGATC CGACGA
3	RRKWYRRRYY RWRWYRRATY YKWYRW
